# Spatial Alignment Facilitates Visual Comparison in Children

**DOI:** 10.1111/cogs.13182

**Published:** 2022-08-16

**Authors:** Yinyuan Zheng, Bryan Matlen, Dedre Gentner

**Affiliations:** ^1^ Department of Psychology Northwestern University; ^2^ STEM Program WestEd

**Keywords:** Spatial alignment, Visual comparison, Structural alignment, Development of visual processing

## Abstract

Visual comparison is a key process in everyday learning and reasoning. Recent research has discovered the spatial alignment principle, based on the broader framework of structure‐mapping theory in comparison. According to the spatial alignment principle, visual comparison is more efficient when the figures being compared are arranged in *direct placement*—that is, juxtaposed with parallel structural axes. In this placement, (1) the intended relational correspondences are readily apparent, and (2) the influence of potential competing correspondences is minimized. There is evidence for the spatial alignment principle in adults’ visual comparison (Matlen et al., 2020). Here, we test whether it holds for children. Six‐ and eight‐year‐old children performed a same‐different task over visual pairs. The results indicated that direct placement led to faster and more accurate comparison, both for concrete same‐different matches (matches of both objects and relations) and for purely relational matches—evidence that the same structural alignment process holds for visual comparison in 6‐ and 8‐year‐olds as in adults. These findings have implications for learning and education.

## 1 Introduction

Analogical comparison, the ability to perceive and transfer relational structure across situations, is an important driver of conceptual learning (Christie & Gentner, 2010; Doumas & Hummel, 2013; Gentner, 2010; Gentner & Hoyos, 2017; Holyoak, 2012). It is especially prominent in education, particularly in mathematics and science (Alfieri et al., 2013; Dixon & Bangert, 2004; Gentner et al, 2016; Goldwater & Gentner, 2015; Goldwater & Schalk, 2016; Hattikudur & Alibali, 2010; Kurtz, Miao, & Gentner, 2001; Novick & Holyoak, 1991; Richland & Simms, 2015; Rittle‐Johnson & Star, 2009; Schunn et al. 2018; Vendetti et al., 2015). One important route of analogical learning is through visual comparison, which is ubiquitous in children's everyday learning as well as in classroom contexts (Franconeri et al., 2012; Gattis, 2002; Matlen et al., 2020). It is a powerful learning process, especially early in development before children have acquired extensive language skills. Visual comparison highlights spatial relational commonalities, aiding children in learning spatial concepts such as symmetry and facilitating transfer between different spatial arrays (Chen, 2007; Christie & Gentner, 2010; Loewenstein & Gentner, 2001; Hribar, Haun, & Call, 2012; Yuan, Uttal, & Gentner, 2017). It has also been shown to aid students in learning mathematical principles and procedures (Begolli & Richland, 2016; Dixon & Bangert, 2004; Hattikudur & Alibali, 2010; Richland & McDonough, 2010; Rittle‐Johnson & Star, 2009) and to support children's learning of scientific principles (Chen & Klahr, 1999; Gentner et al., 2016; Matlen et al. 2011; Nokes‐Malach, et al., 2013). In this paper, we briefly review the literature on visual comparison before turning to a newly discovered principle that facilitates the process of visual comparison––the spatial alignment principle. After reviewing evidence that this principle operates in adults (Matlen et al., 2020), we present two studies testing whether this principle holds for visual comparison in 6‐ and 8‐year‐old children.

### 1.1 Visual comparison and the structure‐mapping theory

Visual comparison has been analyzed using the structure‐mapping framework (Gattis, 2002; Lovett & Forbus, 2017; Markman & Gentner, 1993a; Sagi, Gentner, & Lovett, 2012; Yuan et al., 2017). According to structure‐mapping theory, comparison entails a process of structural alignment based on matching common relational structure (Falkenhainer, Forbus, & Gentner, 1989; Forbus et al., 2017; Gentner, 1983, 2010; Markman & Gentner, 1993a). The idea is that during comparison, people implicitly seek a one‐to‐one mapping in which like relations are aligned and objects are placed in correspondence based on having like roles within the relational structure.

Structural alignment has been widely shown to aid in conceptual abstraction and transfer (Doumas, Hummel, & Sandhofer, 2008; Gentner, 1983, 2010; Gentner & Markman, 1997; Jones & Love, 2007; Kokinov & French, 2003; Krawczyk, Holyoak, & Hummel, 2004; Markman & Gentner, 1993a,b; Sagi et al., 2012; Thibaut, French, & Vezneva, 2010). It supports noticing relational commonalities (Christie & Gentner, 2010; Gentner, Anggoro, & Klibanoff, 2011; Gentner, 2010; Goldwater & Gentner, 2015; Richland & Simms, 2015). For example, Kurtz, Miao, and Gentner (2001) showed undergraduates two different heat‐flow scenarios. Students were more likely to perceive the common underlying causal schema if they compared the two scenarios than if they described each of them separately. Structural alignment also fosters noticing alignable differences (differences that play corresponding roles in two aligned structures) (Gentner & Gunn, 2001; Gentner & Markman, 1997; Markman & Gentner, 1993b; Sagi et al., 2012; Shao & Gentner, 2022).

**Fig. 1:**
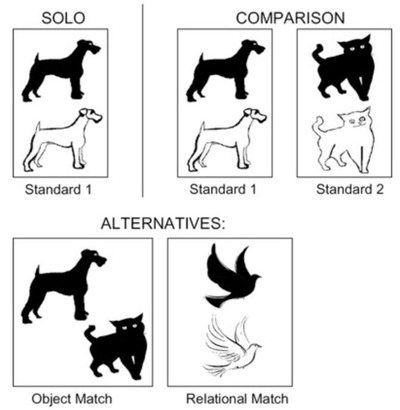
Example of the novel spatial patterns used in Christie and Gentner (2010), depicting a spatial configuration in which a black animal is above the otherwise identical white animal.

In visual figures, the spatial configuration often conveys critical relational information (Gattis, 2002; Lovett & Forbus, 2017; Uttal, 2000). Thus, comparing two visual figures requires aligning their spatial relational structures and placing elements in correspondence based on their having like roles in the common spatial structure (Gentner & Markman, 1997). As with conceptual analogies, structural alignment of spatial structures can reveal commonalities and differences. As an example of how visual comparison highlights spatial commonalities, Christie and Gentner (2010) showed 3‐year‐old children novel spatial patterns—for example, “black thing above white thing, otherwise identical” (Fig. 1). In one study, children were assigned to three conditions: solo, simultaneous comparison, and sequential presentation. In the solo condition, children were told that the figure was a “dax” and were asked to choose another dax from the two alternatives. Children strongly preferred an alternative that shared an object over one that shared the relational pattern: only 9% of 3‐year‐olds’ responses favored the relational match. But when children were given two “daxes” simultaneously and invited to compare them, the results were striking: the comparison group was much more likely to choose the relational match (72% of responses). However, when children were shown the same two standards (with labels) sequentially, their rate of relational responding did not differ significantly from that of the solo group—evidence that comparison is critical to the relational insight.

Visual comparison can also highlight alignable differences. For example, Gentner et al. (2016) used visual comparison to teach 6‐ to 8‐year‐old children the engineering principle that a diagonal brace confers stability. Children were shown two model buildings, one with a diagonal brace (which was, therefore, stable) and one with a horizontal crosspiece, lacking a diagonal brace (which was unstable). The buildings were either highly similar and easy to align or dissimilar and harder to align. When children were asked “Which is stronger” and allowed to wiggle them, they discovered that the stable building barely moved, while the unstable one could be bent nearly to the ground. At this point, all children recognized *which* building was stronger. However, the high‐alignment group was far more likely to discover *why*: they were significantly more likely to spontaneously utilize brace structure in a subsequent task than those who received the low‐alignment pair or no training. Ease of alignment also facilitates difference‐detection in adults: By engaging in comparison, undergraduates are more likely to identify anomalies in complex skeleton structures (Kurtz & Gentner, 2013), and medical students are more likely to detect focal lesions in chest X‐ray images (Kok et al., 2013).

### 1.2 Alignment principles for visual comparison

Given the prevalence and importance of visual comparison both in classroom learning (Alfieri et al., 2013; Begolli & Richland, 2016; Richland & McDonough, 2010; Rittle‐Johnson & Star, 2007, 2009) and in everyday contexts (Gentner et al., 2016; Haryu, Imai, & Okada, 2011; Shao & Gentner, 2022), it is of particular interest to both psychologists and educators to understand what factors prompt comparison and facilitate structural alignment of visual pairs. Three factors that have been identified in prior work are common labels, high overall similarity, and spatiotemporal proximity (Gentner, 2010; Gentner & Hoyos, 2017; Vendetti et al., 2015). We briefly describe these, and then turn to the proposed new factor, spatial alignment.

#### Common labels.

When the same label is applied to different objects, children are likely to compare them in search for commonalities, even when they do not know its meaning (Gentner, 2003, 2010; Gentner & Christie, 2008; Gentner & Namy, 1999). For example, Gentner and Namy (2002) presented 4‐year‐olds pictures from the same categories that also looked similar (e.g., a bike and a tricycle). In the common novel word condition, they were told that both were “blickets.” In the conflicting word condition, they were told that one was a “blicket” and the other was a “daxen.” Next, children were asked to choose which of the two alternatives was of the same kind. One option was from the same category but looked different (a skateboard), whereas the other one was from a different category but looked similar (a pair of round glasses). Hearing the same label led children to select the category match more often than hearing different labels, suggesting that common labels invite alignment that yields deeper commonalities beyond perceptual match. Other research has shown that infants also benefit from common labels in object categorization (Ferry, Hespos, & Waxman, 2010; Fulkerson & Waxman, 2007; Markman, 1989). Together, these findings suggest that common labels invite comparison, allowing children to gain insight into common structure.

#### High overall similarity.

Children (and adults) are more likely to spontaneously engage in comparison between similar items than between dissimilar ones.^1^ Further, high overall similarity facilitates structural alignment (Gentner & Hoyos, 2017; Gentner, Loewenstein, & Hung, 2007; Gentner & Rattermann, 1991; Loewenstein & Gentner, 2001). Overall similarity matches (where object similarity supports the relational alignment) are easier to process than purely relational analogies for both children (Gentner & Toupin, 1986) and adults (Gentner & Kurtz, 2006). Overall similarity is particularly important early in learning, when children's representations are typically rich in objects but sparse in relations. Of course, close comparisons have the drawback that the common structure will typically be more concrete than with more distant comparisons. Distant comparisons (matching in relations but not object features) can support greater generalization—but only if the child can align them. However, studies show that carrying out overall similarity matches can highlight common relations, albeit in a rather concrete frame, and this can facilitate matching more distant pairs that share the same relational structure (*progressive alignment*) (Fyfe et al., 2014; Gentner, Loewenstein, & Hung, 2007; Gentner et al., 2011; Haryu et al., 2011; Kotovsky & Gentner, 1996; Thompson & Opfer, 2010).

#### Spatiotemporal proximity.

There is considerable evidence that children are more likely to compare and align two things if they are spatially and temporally juxtaposed. For example, in Christie and Gentner's (2010) study (described above), young children who compared two juxtaposed exemplars of a spatial pattern were able to abstract and transfer the pattern; but they failed to learn the pattern if the same two exemplars were shown sequentially. Simultaneous presentation has also been shown to improve classroom learning in mathematics (Begolli & Richland, 2016; Rittle‐Johnson & Star, 2009), geoscience (Jee et al., 2014; Matlen et al., 2011), and many other science domains (Alfieri et al., 2013; Gadgil, Nokes‐Malach, & Chi, 2012; Kellman, 2013). For example, Begolli and Richland (2016) taught fifth graders the ratio concept by comparing correct division strategies to misconceptions. Examples were either left visible on the board throughout the presentation or only available sequentially. The results indicated that simultaneous presentation led to better posttest performance and understanding of the ratio principle than sequential presentation.^2^ There is considerable evidence that simultaneous comparison of analogs is advantageous in that it both encourages comparison and supports aligning the relational structures (Christie & Gentner, 2010; Gentner & Hoyos, 2017). It may also lead to reduced cognitive processing load in carrying out alignment (Hyun et al., 2009; Begolli et al., 2018).

### 1.3 Spatial alignment principle

**Fig. 2:**
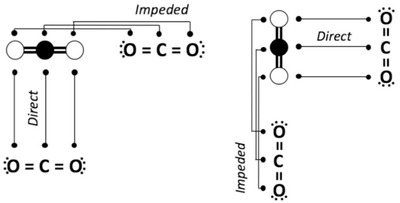
Molecular notations in impeded and direct placement. Adapted from Matlen et al. (2020).

Recently, a previously unexplored factor has emerged as important in visual comparison: *spatial alignment* (Matlen, Gentner, & Franconeri, 2020). The idea is that the comparison process should be more fluent to the degree that (1) the intended relational alignment is readily apparent, and (2) the influence of potential competing correspondences is minimized. By the same reasoning, visual comparison should be impeded when the placement of visuals leads to intervening potential correspondences between the correctly corresponding elements. More specifically, this principle states that visual comparison is more fluent when the figures are placed orthogonally to their structural axes (i.e., the axes along which the main relations apply).^3^ Fig. 2 depicts two examples of molecular notations. In the left panel, the molecule forms an ABA pattern and has a horizontal orientation—in other words, the main relational pattern is presented horizontally. A comparison between the molecule depiction and its notation is facilitated if they are laid out vertically, and impeded if they are laid out horizontally. The reverse is true in the right panel, where the same molecular structure is presented in a vertical orientation (i.e., it has a vertical structural axis).

**Fig. 3:**
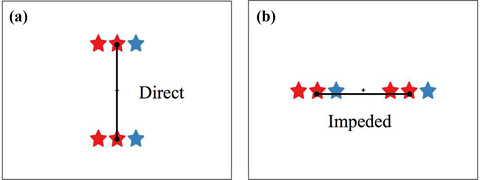
Color triplets with horizontal structural axes in direct and impeded placement.

What are the advantages of orthogonal placement? In *direct placement*, the figures are juxtaposed and orthogonally placed. This means that spatially corresponding elements and relations are juxtaposed and relatively far from competing noncorresponding elements. This makes the relational alignment maximally easy to notice. In contrast, in *impeded placement*, the figures are placed consecutively along their structural axes. In this placement, other potential correspondences intervene between the correctly corresponding elements. As a result, the correct structural alignment may take longer to discover or be missed altogether. Fig. 3 illustrates this principle with example pairs used by Matlen et al. (2020), as well as in the current experiment. Here, all triplets have horizontal orientations (i.e., horizontal structural axes). Direct placement is shown in Fig. 2A, where these triplets are placed vertically, and impeded placement is shown in Fig. 2B, where the triplets are placed side by side. According to the spatial alignment principle, processing a visual comparison should be more efficient in direct placement than in impeded placement.

To test this prediction, Matlen et al. (2020) conducted a same‐different judgment task in adults. Participants saw pairs of triplets and were asked to identify whether the pair was the same or different as fast and accurately as possible. For example, *red‐red‐blue* has a different pattern than *red‐blue‐red*, so the response should be “different.” The orientation and layout of triplets were systematically manipulated to create direct and impeded comparisons for both horizontal and vertical triplets. As predicted by the spatial alignment principle, people were both faster and more accurate on direct trials than on impeded trials. These result patterns were found for triplets varying in shape (e.g., square‐square‐triangle) as well as those varying in color.

In a further study, Matlen et al. asked whether the above results could be attributed to simple object‐matching, as opposed to structural alignment. To test this, they paired together shape triplets and color triplets to form relation‐only trials (Fig. 5). Participants were instructed to press “same” if the relational pattern was the same. For example, they were told that *square‐triangle‐square* has the same relational pattern as *red‐blue‐red*, so they should press “same” for pairs like this. The results again supported the spatial alignment principle, with faster performance on direct trials than on impeded trials. The results also showed that participants were slower on relation‐only trials (i.e., cross‐dimension trials) than on object+relation trials (i.e., within‐dimension trials) (although there was no significant difference in accuracy). This is consistent with prior findings that comparison is in general easier when there exist object correspondences as well as relational correspondences (Gentner & Hoyos, 2017; Gentner & Rattermann, 1991; Gentner & Toupin, 1986). Overall, the findings provide strong support for the spatial alignment principle, and for structure‐mapping more generally.

Interestingly, Matlen et al. also found an interaction between triplet orientation and placement. In particular, the increase in response time for impeded (relative to direct) trials was lower for horizontal pairs than for vertical pairs. This lower impedance cost for horizontal pairs, termed the *horizontal advantage*, held across both object+relation trials and relation‐only trials. This suggests that horizontal spatial patterns may be encoded more robustly and/or faster, making the alignment process less vulnerable to the adverse effect of impeded placement. Matlen et al. speculated that this horizontal advantage could be due to greater encoding fluency for horizontal patterns than for vertical patterns—perhaps due to reading experience (Thibaut, French, & Vezneva, 2010), which could lead English readers to be more fluent at encoding information from left to right than from top to bottom.

### 1.4 Does the spatial alignment principle hold for children?

Our chief goal in this work is to discover to what extent the spatial alignment principle operates in children. An answer to this question will shed light on the development of visual comparison ability and provide educators with a better understanding of how to design instructional materials. Because children are highly reliant on perceptual supports for structural alignment, our expectation is that spatial alignment will be at least as important in children's comparison as in adults’.

As a secondary goal, we further explored Matlen et al.’s (2020) speculation concerning the finding of horizontal advantage, that adults showed a lower impedance cost for horizontal figures than for vertical figures. Matlen et al. suggested that extensive practice in reading may lead to greater fluency in encoding horizontal patterns (see also Thibaut et al., 2010). If this explanation is correct, then this asymmetry between horizontal and vertical figures should be attenuated or lacking in children for whom reading has not yet become a fluent skill.

Children start to form print awareness at age 4, including recognizing that print is read from left to right (Hiebert, 1981; Justice & Ezell, 2001). Reasoning that this habit of left‐to‐right scanning should become stronger with habitual use, we targeted 6‐ and 8‐year‐old children in our studies. If Matlen et al.’s speculation is correct, then this pattern of horizontal advantage should be missing or greatly diminished in 6‐year‐olds, who are just learning to read, and possibly greater in 8‐year‐olds, who have had more practice with reading. Further, the degree of horizontal advantage should be related to reading proficiency.

We adapted the procedures used in Matlen et al. (2020) to be child friendly and tested the spatial alignment principle over two age groups: 6‐year‐olds (study 1) and 8‐year‐olds (study 2). We hypothesized that the spatial alignment principle should apply to both age groups, but that (if found) the horizontal advantage would be more prominent in older children. As in the adult study, participants were asked to provide same‐different judgment for pairs of triplets. The test pairs consisted of either color or shape triplets in the first two blocks (within‐dimension blocks with object+relation matches) or a combination of color and shape triplets in the last block (cross‐dimension block with relation‐only matches). Pairs systematically varied in triplet orientation and placement.

To prepare them for the task, children were given extensive training (see study 1 procedure). In addition, we used a fixed order of blocks, such that the cross‐dimension block was always given after the two within‐dimension blocks. This is based on findings that children find purely relational matches harder to process than concrete object+relation matches (Gentner & Rattermann, 1991; Gentner & Toupin, 1986; Haryu et al., 2011; Kotovsky & Gentner, 1996). By running the cross‐dimension trials last, we ensured that children were well‐practiced in the basic task before they encountered the more difficult cross‐dimension trials.

We had three predictions. First, and most importantly, we predicted that both 6‐ and 8‐year‐old children's performance would follow the spatial alignment principle. That is, children should be faster and/or more accurate in visual comparison for direct placement—in which corresponding elements and relations are juxtaposed and far from competing matches—than for impeded placement, in which there are spatially close competing matches. Consistent with structure‐mapping theory, this prediction should hold both for within‐dimension (overall similarity) comparisons and for cross‐dimension (purely relational) comparisons. Our second prediction, also following structure‐mapping theory, is that children's performance should be better on within‐dimension (object+relation) trials than on cross‐dimension (relation‐only) trials.^4^ This would parallel the finding that adults in Matlen et al.’s study were faster on object+relation trials than on relation‐only trials.

Our third prediction was that, as in Matlen et al. (2020), there should be an interaction between placement and dimensionality whereby the advantage of direct over impeded placement would be larger for object+relation trials than for relation‐only trials. Matlen et al. hypothesized that this comes about because object matches are highly salient and faster to process than relational matches (Gentner & Kurtz, 2006; Goldstone & Medin, 1994). In direct placement, even though a purely relational alignment is fairly easy to notice, having matching objects offers an additional basis for quickly judging same or different besides relations. But in impeded placement, the interference is purely relational in relation‐only trials, whereas for object+relation trials, the intervening object matches lead to additional interference (Fig. 3). Thus, we expected the advantage of direct over impeded placement to be larger for object+relation trials than for relation‐only trials for both age groups.

Beyond testing the above three predictions, a further goal of this research was to explore whether children would show a horizontal advantage—that is, lower impedance cost for horizontal figures than for vertical figures—and if so, whether this would be related to their reading fluency. If, as Matlen et al. (2020) conjectured, robust horizontal encoding is the result of experience—especially practice in reading—then (1) this pattern should be more pronounced in 8‐year‐olds than in 6‐year‐olds, and (2) if a horizontal advantage does appear, it should be related to reading ability. We measured reading fluency in children through two standard reading assessments, word reading fluency (WRF) and oral reading fluency (ORF) tests, adapted from Dynamic Indicators of Basic Early Literacy Skills (DIBELS; University of Oregon, 2018; Deno & Mirkin, 1977; Good & Kaminski, 2002, 1996). DIBELS, developed by education experts at University of Oregon, is a reading assessment tool for students from kindergarten to grade 8 that is widely used in schools (About Us: Our History, 2019). The assessments are argued to be valid and reliable (Good & Kaminski, 1996) and effective at capturing students’ reading progress (Kaminski et al. 2008). The two tests, WRF and ORF, assess reading level by measuring the number of words children read correctly in 1 min.

We next present the studies—study 1 (6‐year‐old children) and study 2 (8‐year‐old children).

## 2 Study 1

### 2.1 Methods

#### 2.1.1 Participants

Twenty‐nine 6‐year‐old children participated (*M* = 6.51 years, *Range =* 6.00–7.06 years, 11 females; among the families who indicated race, 79% were White, 11% were Asian, 7% were multiracial, and 3% indicated other races and ethnicities). For the 28 families who indicated parental educational level, all except one indicated college degree or higher. One additional child was recruited but excluded due to failure to complete the experiment. Children were recruited through a database of families willing to participate in child research from a large Midwestern city. All families had English as the primary language.

Following Matlen et al. (2020), the sample size was planned for our main hypothesis of spatial alignment, based on a repeated‐measures analysis of variance (ANOVA) specifying a within‐between interaction (2 triplet orientations × 2 placements). Because of the within‐subject design, to detect a medium effect of Cohen's *f* of 0.4 requires a sample size of 24, assuming error probability of 0.05 and power of 0.80. Twenty‐nine participants were run because multiple families were recruited at the same time. This sample size was not powered to detect a between‐subject difference in reading fluency, but if a large effect was present, it could be revealed by our exploratory analysis.

#### 2.1.2 Materials and design

**Fig. 4:**
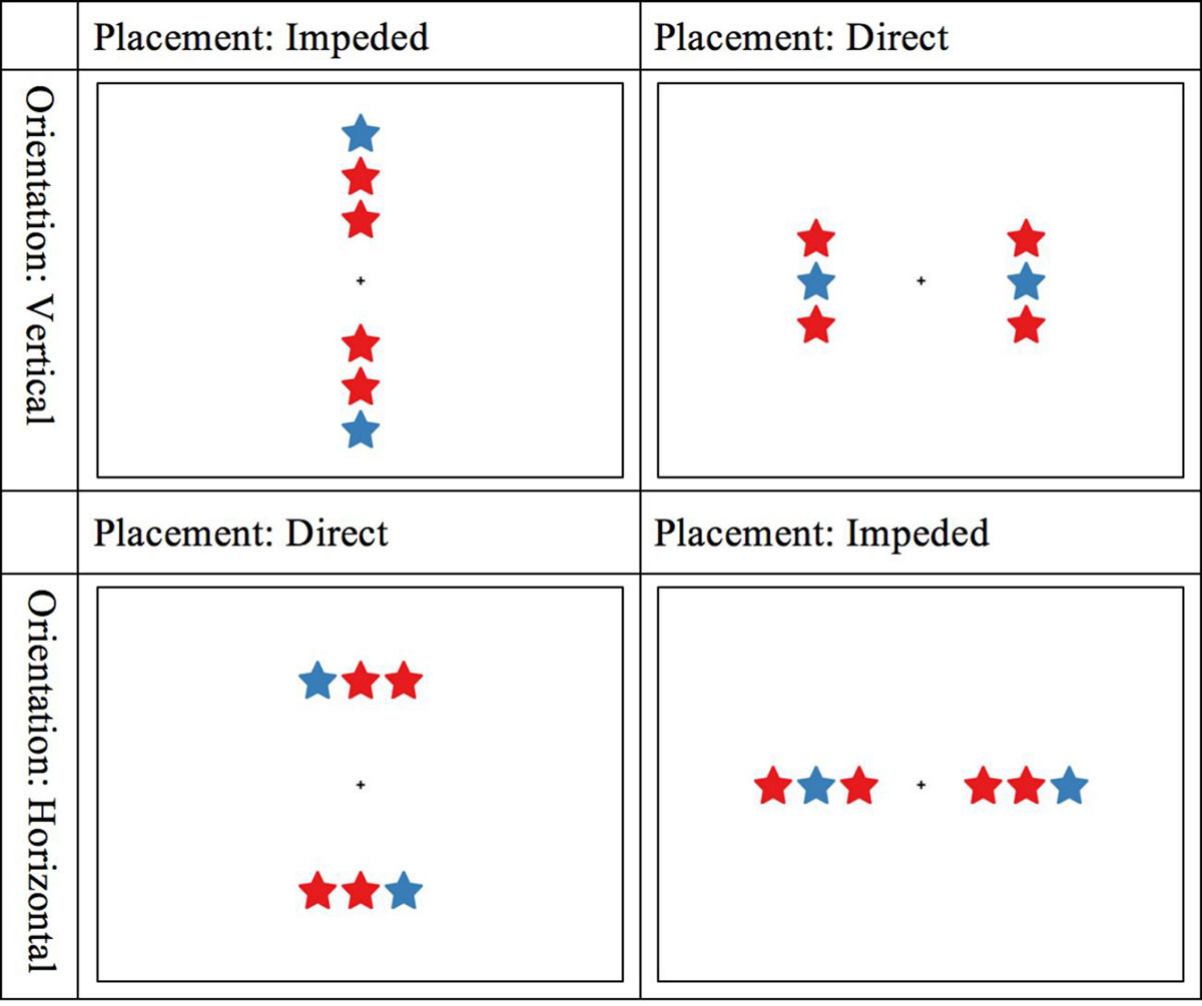
Examples of color‐color test trials in different placements, orientations, and concordance (same or different).

**Fig. 5:**
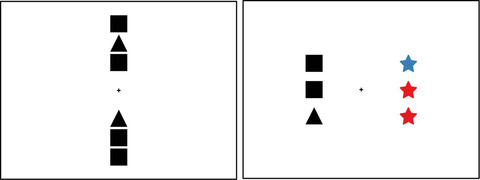
Examples of within‐dimension versus cross‐dimension triplets, showing a shape‐shape test trial on the left and a color‐shape test trial on the right.

Stimuli were adapted from Matlen et al.’s (2020) experiments 1 and 3. Children were shown a pair of triplets and either gave a same‐different judgment (for within‐dimension pairs) or responded as to whether the relational patterns were the same or different (for cross‐dimension pairs). We used a fixed order presentation where the within‐dimension blocks were given first, followed by the cross‐dimension block. This was done to allow children to practice with the more straightforward same‐different task before engaging in the purely relational judgment. Figs. 4 and 5 depict the types of triplets used in the studies. Three blocks of triplet pairs were used (color‐color, shape‐shape, and color‐shape; referred to as *Dimension*). Pairs varied in the structural axes of triplets (both vertical or both horizontal; referred to as *Orien*tation) and relative placement between the two triplets (direct or impeded; referred to as *Placement*). Pairs depicted either the same or different patterns (referred to as *Concordance*). The design was within‐subject and consisted of 2 Dimension (within vs. cross) × 2 Orientation (horizontal vs. vertical) × 2 Placement (direct vs. impeded) × 2 Concordance (same vs. different). Each within‐dimension block contained 36 test trials and four catch trials, and the last cross‐dimension block contained 72 test trials and eight catch trials. In total, participants were required to complete 160 trials in the test phase in addition to the trials used in training. Table 1 shows a summary of this terminology.

**Table 1:** Table of definition for the terms used in our studies

Term	Definition
Dimension (within or cross)	The type of triplet pairs (color‐color/shape‐shape or color‐shape)
Orientation (horizontal or vertical)	The direction of the structural axis of a triplet
Placement (direct or impeded)	The relative placement between a pair of triplets
Concordance (same or different)	Whether the pair of triplets share the same or different patterns

##### 2.1.2.1 Within‐dimension triplets

In *color‐color* trials, each triplet consisted of star shapes of two colors (two reds [rgb: 228, 26, 28] and one blue [rgb: 55, 126, 184]). In *shape‐shape* trials, each triplet consisted of three black geometric shapes (two squares and one triangle; see Figs. 4 and 5). Using color trials as an example, the sequence of colors within each triplet was fully counterbalanced, resulting in three possible orderings for a triplet [*blue‐red‐red, red‐blue‐red*, or *red‐red‐blue*] and nine [3×3] possible pairings for a pair. A “same” response was called for when triplets within a pair were identical. Because of the fully counterbalanced orderings across triplets, one‐third of all test trials were “same” and the rest “different.” The same was true for shape trials.

##### 2.1.2.2 Cross‐dimension triplets

A similar plan was used for cross‐dimension triplets. In color‐shape trials, one triplet consisted of stars of reds and blue and the other of black squares and triangle. Fig. 5 shows an example of color‐shape test trials. For color‐shape trials, one triplet had three possible orderings of color [*blue‐red‐red, red‐blue‐red*, or *red‐red‐blue*] and the other one had three possible orderings of shape [*triangle‐square‐square, square‐triangle‐square*, or *square‐square‐triangle*]. In addition, another factor of 2 was needed to account for whether the color triplet or the shape triplet occurred first (to occur first means being on the left or at the top). This resulted in 18 possible pairings [3×3×2], twice as many as those in each within‐dimension block. A “same” response was called for when triplets within a pair contained the same pattern. Having the same patterns required matching on first‐order relation and, therefore, spatial correspondence for the unique element within each triplet (e.g., *blue‐red‐red* shares the same pattern with *triangle‐square‐square* but not with *square‐triangle‐square* or *square‐square‐triangle*). One‐third of all test trials were “same.”

We systematically varied the orientation and placement of each pair and created four possible combinations (2 Orientation: horizontal vs. vertical × 2 Placement: direct vs. impeded). Triplets within a pair always shared the same orientation of their structural axes, but their placement varied. In *direct* trials, the placement of triplets was orthogonal to their structural axes (e.g., horizontal triplets placed vertically). In *impeded* trials, triplets were placed consecutively along their structural axes (e.g., horizontal triplets laid out horizontally). Together with the number of possible pairings, our manipulation resulted in 36 [9×4] test trials for each within‐dimension block and 72 [18×4] test trials for the cross‐dimension block.

Triplets were presented on a 13‘ monitor at 1024×768 resolution. Displays centered at a black fixation cross (16×16 pixels in width and height) on a white background. Each triplet element had a 74×74 bounding box. For within‐dimension triplet, elements were 10 pixels away from each other, resulting in the size of 74×242 pixels for vertical triplets and 242×74 pixels for horizontal triplets. For cross‐dimension triplet, elements were 40 pixels apart. Centers for all triplets were 205 pixels away from the fixation.

#### 2.1.3 Procedure

After obtaining parental consent and children's verbal assent, the experimenter brought participants into a quiet room with a 13‐inch MacBook Pro open. There were four experimental phases: key orientation, same‐different training, testing, and reading assessment, with the first three phases run on the software platform of PsychoPy3 (v3.0.6; Peirce et al., 2019) and the last one on paper. Each phase was described in detail below.

##### 2.1.3.1 Key orientation phase

The goal of this phase was to familiarize children with the key pressing required for the testing phase. The three keys participants were asked to press throughout the experiment were “x,” “comma,” and spacebar. The first two keys were labeled as “S” and “D” on white stickers with order counterbalanced across participants, and the spacebar was always labeled as “Next.” The experimenter demonstrated how to put the two index fingers on “x” and “comma” and both thumbs on the spacebar to press the keys. After children put their hands on the keyboard, they were invited to press “Next” to see a picture and were instructed to press the left‐hand key (“x”) if a letter “L” showed up on the left side of the screen and the right‐hand key (“comma”) if a letter “R” showed up on the right side of the screen. The trial began after children press the “Next” key. A fixation cross first appeared for 1000 ms, after which the picture appeared with the fixation cross still at the center. This presentation format was used throughout the experiment. This was repeated for six random trials, and after the third trial, children were prompted to respond as fast as they could. During training, automated feedback appeared on the screen after each response—“Great Job!” for the correct answer and “Whoops!” for the wrong answer.

##### 2.1.3.2 Same‐different training phase

**Fig. 6:**
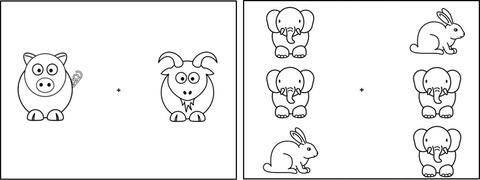
Example trials used in the same‐different training phase. The pair on the left was used on trial 1 and the one on the right was used on trial 5.

The goal of this phase was to provide a series of training steps so that children would understand the task. Children were told to press “S” if they think the two pictures were the same and “D” if they were different. Automated feedback was provided after each response. There were eight trials in a fixed order, alternating between different and same trials and between vertical and horizontal placement. The first four trials required children to do a same‐different match on two animals (i.e., pig and goat). The last four trials showed pairs of animal triplets in direct placement (consisting of two elephants and a rabbit) (Fig. 6). On the first triplet trial (i.e., trial 5), the experimenter told children that instead of one animal at a time, they were going to look at patterns. The experimenter then named the pattern on the left (*elephant‐elephant‐rabbit*), let children name the pattern on the right, and asked them whether the triplets contained the same or different patterns. Depending on children's response, the experimenter said “Yes/ no. They have different patterns even though they have the same animals.” Additional explanation was provided if children were wrong: “Here (pointing to the left triplet) the rabbit is at the bottom but here (pointing to the right triplet) the rabbit is at the top. The animals are in different order. And that's why they don't have the same patterns, even though they have the same animals.” Children were asked to do the rest on their own.

##### 2.1.3.3 Testing phase

The testing phase was divided into three blocks as detailed above: The first two were object+relation, within‐dimension (colors or shapes, with presentation order randomized) and the last one was always relation‐only, cross‐dimension (i.e., shape‐color). Children were instructed to respond as fast as they could while also trying to get all trials correct. Within each block, children first completed randomized practice trials with feedback provided (three for within‐ and six for cross‐dimension blocks). If children made no error, they proceeded to the test trials after the experimenter checked for whether they had any questions. No feedback was provided for the test trials. If children erred on any practice trials, they were asked to redo the practice trials for that block up to three times to ensure understanding of the task instructions. For within‐dimension blocks, no children erred more than three times. All triplet pairs in the practice contained different elements from those in the actual experiment. Shape practice trials consisted of octagons and stars; and color practice trials consisted of black and white stars. Both direct and impeded placements were included in practice trials.

For cross‐dimension practice trials, the pairs contained circle and squares for the shape triplets and one red and two blue stars for the color triplets. Special instructions were given on the first practice trial. The experimenter paused and explained to children that they would need to match locations of the unique element in each triplet instead of relying on identical patterns. Specifically, the experimenter read the patterns of triplets on the screen. For example, if the pair depicted *blue‐red‐blue* and *square‐circle‐square*, he would say, “here (pointing to the first triplet) the red star is different from the two blue stars and this different thing is in the middle. Here (pointing to the second triplet) the circle is different from the two squares and this different thing is also in the middle. So they have the same patterns.” If children erred on any practice trial, they were asked to redo the practice up to three times. The same explanation was repeated on the first practice trial, but in a more engaging fashion—for example, by checking with children for understanding throughout the explanation. All but one child passed the cross‐dimension practice within three tries. Data of this child for the cross‐dimension block were excluded from further analyses.

There were 36 test trials for within‐ and 72 test trials for cross‐dimension blocks, with trial order randomized within each block. Each trial began with an instruction screen that wrote “Ready? Press ‘Next’.” After participants pressed the space bar, a fixation cross appeared for 1000 ms, following which a pair of triplets showed up. Participants indicated whether the triplets shared the same or different patterns by pressing “S” and “D,” respectively. Response was timed after stimulus onset and no feedback was provided. After each response, the instruction screen for the next trial appeared. After completing the first block, the experimenter told children that they were going to play the same game but with new pictures. After completing the second block, the experimenter told children that the same pattern matching game was made harder, and he would explain why it was harder during the practice.

Immediately after all test trials in each block, catch trials were included to detect children who might be responding randomly due to fatigue or failure to conform to the instructions. There were four catch trials for each within‐dimension block. In these trials, the triplets in a pair were in direct placement and contained only a single color (e.g., *blue‐blue‐blue* and *blue‐blue‐blue*) or shape (e.g., *square‐square‐square* and *square‐square‐square*). The cross‐dimension catch trials consisted of eight directly placed pairs of triplets that contained a single color and shape (e.g., *blue‐blue‐blue* and *square‐square‐square*). Catch trials varied in triplet orientation (horizontal or vertical) and in element value (blue and red for colors and square or triangle for shapes), but all elements came from the test trials of the same block. The exclusion criterion was predetermined to be more than one error for each within‐dimension block and more than two errors for the cross‐dimension block.

To motivate children to stay focused on the task, before the first test block began, children were told that they could earn medals in this game to exchange for prizes, and the more they played, the more medals they could earn. The three medals were bronze, silver, and gold medals, earned during the first, second, and third blocks, respectively. Unbeknownst to them, all children received rewards at the end. The computer task took about 30 min to finish.

##### 2.1.3.4 Reading assessment phase

Two reading tests were used (WRF and ORF). We used the materials intended for first‐grade students because we focused on 6‐year‐olds. Following the instructions and requirement specified by DIBELS (University of Oregon, 2018), in the WRF test, children were given a sheet that contained a list of words and asked to read the words clearly from left to right and top to bottom. They were told that if they got stuck on a word, the experimenter would tell them the word so they could keep going. The experimenter read the word for them only if they were stuck for 3 seconds, and did not correct any mispronounced words otherwise. The experimenter timed the session secretly and stopped children after 60 seconds. Afterward, the experimenter gave children another sheet of paragraphs for ORF and told them to do the same thing. As prescribed in the DIBELS 8th Edition Administration and Scoring guide (2019), WRF and ORF scores were computed by counting the number of correctly read words in 1 min. Twenty‐five of the 29 participants completed the two reading assessments. Data of one child were excluded due to experimenter error.

### 2.2 Results

All analyses were conducted in R (Version 3.4.1; R Core Team, 2015). The two main variables of interest were accuracy and response time for the test trials. For the main analyses, we excluded any trial whose response time was three standard deviations longer than the mean response time with respect to each subject and block dimensionality (within‐ or cross‐dimension).^5^ This constituted 1.8% of all trials. In addition, response time was only analyzed for correct trials. Data in a block were also excluded if participants failed the catch trials for that block based on the above criteria. Overall, one participant failed the color block, two participants failed the shape block, and three participants failed the color‐shape block.

**Fig. 7:**
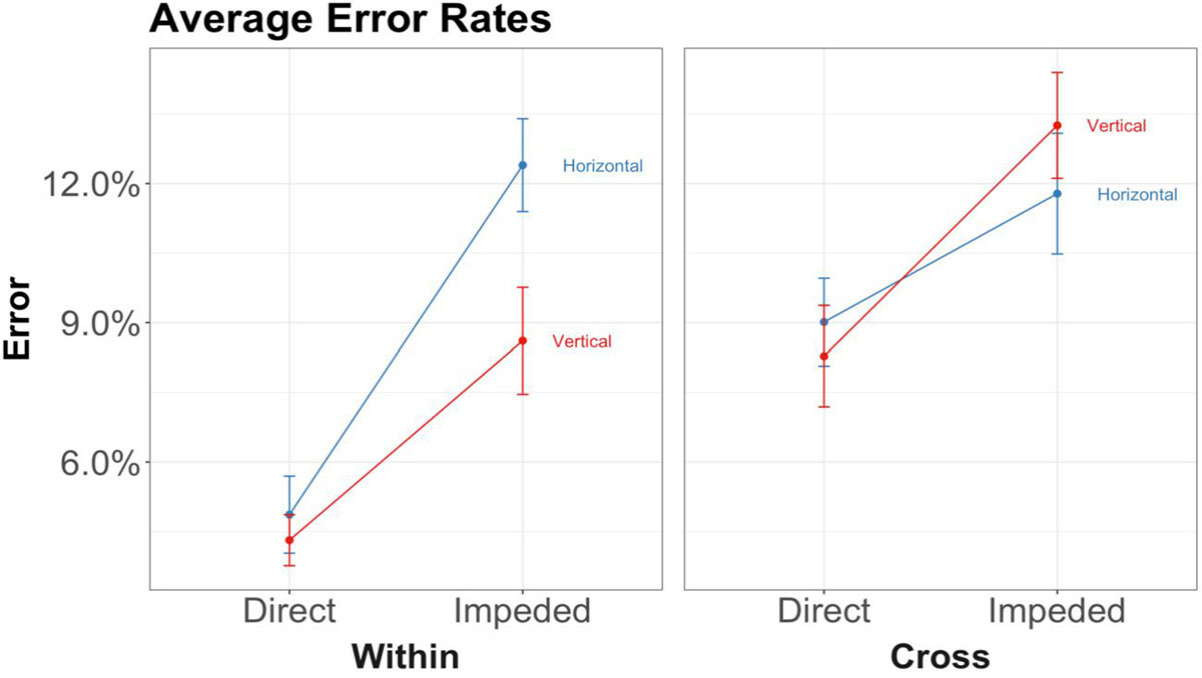
Results of study 1: Error rates by triplet orientation, placement, and whether the block is within‐dimension or cross‐dimension. Error bars represent within‐subject standard errors.

**Fig. 8:**
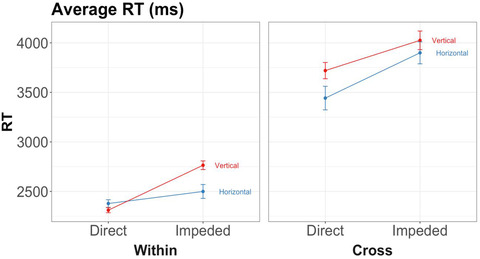
Results of study 1: Response times by triplet orientation, placement, and whether the block is within‐dimension or cross‐dimension. Error bars represent within‐subject standard errors.

To preview, the main results bore out our first two predictions—(1) that 6‐year‐olds would be faster and/or more accurate on trials in direct placement than on trials in impeded placement (the spatial alignment principle) and (2) that children would perform better on within‐dimension trials than on cross‐dimension trials (Figs. 7 and 8). Before presenting the planned comparisons testing these predictions, we first present an overall analysis in order to explore other potentially significant main effects or interactions. (We will return later to our third prediction, that the advantage of direct placement should be higher for within‐dimension trials than for cross‐dimension trials.)

For the overall analysis, we conducted 2 (Placement) × 2 (Dimension) × 2 (Orientation) × 2 (Concordance) repeated‐measures ANOVAs for error rates and response times, with subject as the error term. Definitions of the terms are summarized below (see also Table 1):

- *Dimension*: within or cross, referring to the triplet types (color‐color/shape‐shape or color‐shape)
- *Orientation*: vertical or horizontal, referring to the structural axes of triplets
- *Placement*: direct or impeded, referring to the relative placement of the two triplets
- *Concordance*: whether the two triplets share the same or different patterns

To calculate error rates and response times, we averaged the raw scores based on the mean in all trials with respect to each variable of interest for each participant. For the ANOVAs, the variables of interest are Placement, Dimensionality, Orientation, and Concordance. Descriptive statistics and graphs of error rates and response times are shown in Table 2 and Figs. 7 and 8.^6^

**Table 2:** Results of study 1: Means and standard deviations for error rates and response times on test trials by dimensionality and placement

		Error rates		RT (in ms)	
Dimensionality	Placement	*M*	*SD*	*M*	*SD*
Within	Direct	4.61%	0.08	2350	623
	Impeded	10.9%	0.13	2622	855
Cross	Direct	8.70%	0.14	3526	2025
	Impeded	12.6%	0.17	3892	2108

#### 2.2.1 Effects of spatial alignment and dimensionality

##### 2.2.1.1 Error rates

The repeated‐measures ANOVA on error rates revealed only a significant main effect of placement, *F*(1, 380) = 24.10, *p* < .001, *ηp* ^2^ = 0.06. There was no significant effect of dimensionality, *F*(1, 380) = 3.33, *p* = .07, *ηp* ^2^ = 0.01, nor any significant interaction.

Planned paired *t*‐tests^7^ were carried out for our first two predictions concerning the effects of placement and dimensionality. The first was that children should be faster and/or more accurate in visual comparison for direct placement than for impeded placement. The results bore out this prediction—children made significantly more errors on impeded trials (*M* = 0.12, *SD* = 0.15) than on direct trials (*M* = 0.07, *SD* = 0.11), *t* = 5.15, *p* < .001, *d* = 0.38, 95% CI = 0.23–0.53. Our second prediction was that children should be faster and/or more accurate on within‐dimension trials than on cross‐dimension trials. In this case, the difference in error rates between cross‐dimension trials (*M* = 0.11, *SD* = 0.16) and within‐dimension trials (*M* = 0.08, *SD* = 0.11) was not statistically significant, *t* = 1.93, *p* = .055, *d* = 0.16, 95% CI = −0.004 to 0.33.

##### 2.2.1.2 Response time

The repeated‐measures ANOVA on response times revealed main effects of placement, *F*(1, 380) = 11.65, *p* < .001, *ηp* ^2^ = 0.03, and dimensionality, *F*(1, 380) = 200.30, *p* < .001, *ηp* ^2^ = 0.35. There was no significant interaction effect.

Planned paired *t*‐tests showed that, consistent with Prediction 1, children were faster on direct (*M* = 2904, *SD* = 1573) than on impeded trials (*M* = 3221, *SD* = 1695), *t* = 4.59, *p* < .001, *d* = 0.19, 95% CI = 0.11–0.28. Prediction 2 was also borne out: children were faster on within‐dimension trials (*M* = 2486, *SD* = 758) than on cross‐dimension trials (*M* = 3709, *SD* = 2070), *t* = 11.65, *p* < .001, *d* = 0.71, 95% CI = 0.58–0.85.

Taken together, the results confirm our first hypothesis: children were both faster and more accurate on trials in direct placement than on trials in impeded placement (Prediction 1). The results also bore out our second hypothesis: children were faster, though not more accurate,^8^ on within‐dimension (relation+object) trials than on cross‐dimension (relation‐only) trials (Prediction 2).

#### 2.2.2 Interaction between spatial alignment and dimensionality

Our third main prediction was that the advantage of direct over impeded placement would be greater for within‐dimension trials than for cross‐dimension trials. The rationale is that the salient object matches should facilitate performance in direct placement, but interfere with performance in impeded placement; this will not apply to cross‐dimension trials, which do not involve the same objects.

**Fig. 9:**
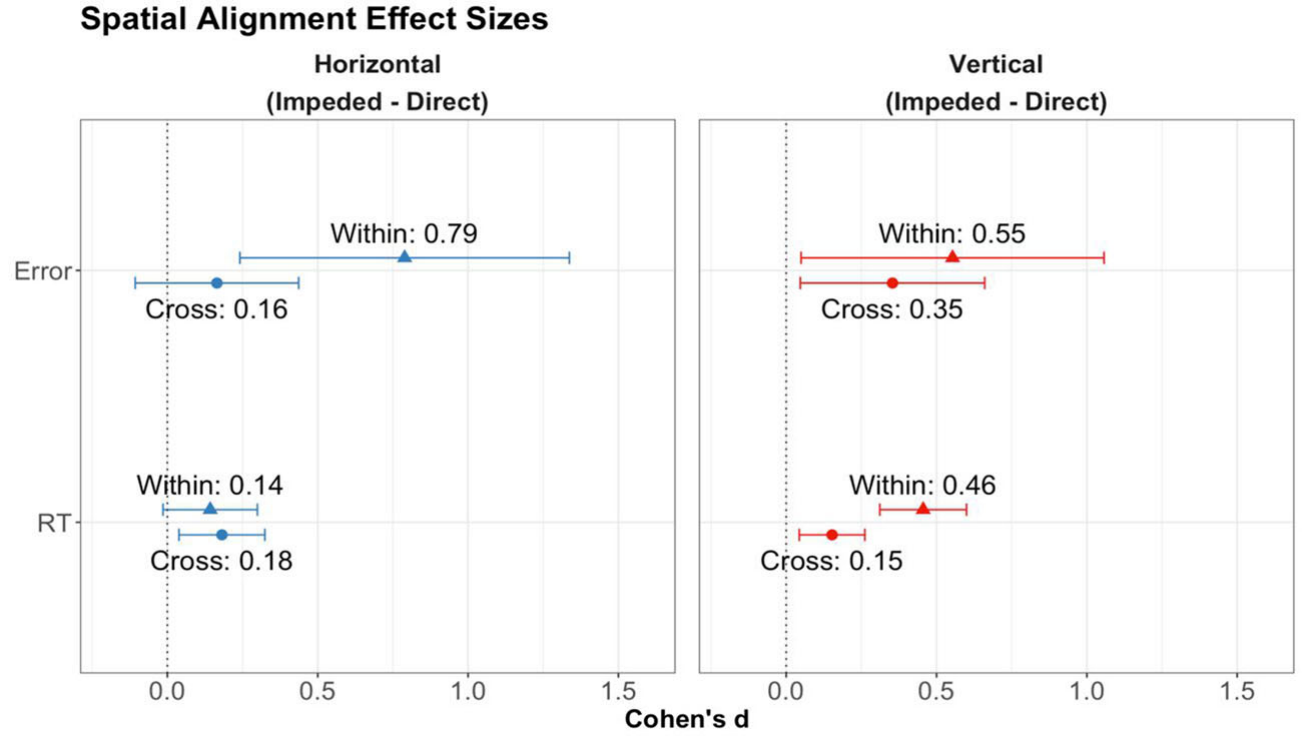
Results of study 1: Effect sizes for error rates and response times by triplet orientation, placement, and whether the block is within‐dimension or cross‐dimension. Error bars represent confidence intervals.

To test this prediction, we compared the effect sizes of error rates and response times between direct and impeded placement for both within‐ and cross‐dimension trials. Effect size (Cohen's *d*) is used instead of simple mean because blocks with different dimensionality might have different means and standard deviations, which is better addressed by an effect size measure. For this analysis, we combine horizontal and vertical triplets, because our third prediction applies to both orientations and because the ANOVA models above did not reveal any effect of orientation. However, for clarity, we present separate graphs for horizontal and vertical triplets (Fig. 9). The detailed analysis of picture orientation is reported in the section of horizontal advantage below. Table 3 and Fig. 9 summarize the statistics.

**Table 3:** Results of study 1: Effect size estimates for the advantage of direct over impeded placement

	Within		Cross	
Measure	*d*	*95% CI*	*d*	*95% CI*
Error rates	0.70	0.27, 1.13	0.27	0.06, 0.49
RT	0.32	0.19, 0.45	0.18	0.10, 0.25

For both error rates and response times, Cohen's *d*s appeared larger for within‐dimension trials than for cross‐dimension trials. However, the overlap in confidence intervals suggests that the differences between trial types were not significant. Therefore, the current data were consistent with but did not provide statistical support for our third prediction, that the advantage of direct over impeded placement would be larger for object+relation matches than for relation‐only matches.

#### 2.2.3 Horizontal advantage

We next turn to our exploratory questions of whether 6‐year‐olds, like adults, would show a horizontal advantage, and whether this would be related to reading fluency. As discussed above, our results (like those of Matlen et al., 2020) show an *impedance cost*—that is, response times were slower for impeded than for direct trials. However, Matlen et al. further found a *horizontal advantage—*that is, the impedance cost for response time was lower for horizontal triplets than for vertical triplets. We next ask whether children also show this horizontal advantage.

To arrive at the degree of horizontal advantage, for each child, we first subtracted the response times of direct trials from those of impeded trials, for triplets of both horizontal and vertical orientations (paired on other variables; see Footnote 8). A positive score indicates a direct‐over‐impeded advantage; the smaller it is, the lower the impedance cost is. If children mirror the horizontal advantage found in adults, they will show a lower impedance cost for horizontal than for vertical figures. Paired *t*‐tests showed that for within‐dimension trials, the direct‐over‐impeded advantage (i.e., the impedance cost for response time) was significantly smaller for horizontal triplets than for vertical triplets (*M_Horizontal_* = 111, *SD_Horizontal_* = 321, vs. *M_Vertical_* = 448, *SD_Vertical_* = 356), *t* = 5.55, *p* < .001, *d* = 0.99, 95% CI = 0.55–1.43. For the cross‐dimension trials, the magnitude of the direct‐over‐impeded advantage did not differ between horizontal and vertical triplets (*M_Horizontal_* = 353, *SD_Horizontal_* = 685, vs. *M_Vertical_* = 348, *SD_Vertical_* = 615), *p* = .98. These results are consistent with there being a horizontal advantage for within‐dimension, but not cross‐dimension, trials.

However, before drawing conclusions, we must consider children's error rates on within‐dimension trials. Using the same procedure, we calculated the impedance cost for error rates (i.e., error rates for impeded placement minus error rates for direct placement). The impedance cost for horizontal triplets (*M_Horizontal_* = 0.076, *SD_Horizontal_* = 0.122) was nonsignificantly higher than the impedance cost for vertical triplets (*M_Vertical_* = 0.043, *SD_Vertical_* = 0.097). Although this difference was not statistically significant in a *t*‐test, *p* = .22, we cannot rule out the possibility of a speed‐accuracy tradeoff. Thus, we cannot conclude that 6‐year‐old children showed a horizontal advantage in response time. However, it remains possible that across individuals, there could be a correlation between reading ability and a horizontal advantage in response time.

#### 2.2.4 Horizontal advantage and reading fluency

Matlen et al. speculated that the horizontal advantage found in adults might reflect robust horizontal encoding resulting from reading experience. If so, we would expect to see a larger horizontal advantage in children with higher reading scores in a regression analysis. (Recall that reading fluency was assessed by two standard tests, as described above).

On average, children read 18.62 (*SD* = 11.55) correct words in the WRF test and 32.96 (*SD* = 36.15) correct words in the ORF test (see Appendix A). The large standard deviations indicate high variability in reading skills. Performance on the two tests was highly correlated, *r* = .84, *p* < .001, so we combined the WRF and ORF scores into a composite score by calculating the weighted sum of the two scores for each participant [WRF * 0.1351 + ORF * 0.2536] based on the weights intended for first‐grade children (DIBELS Scoring Guide, 2019). The resulting reading score had a mean of 10.87 and a standard deviation of 10.62.

**Fig. 10:**
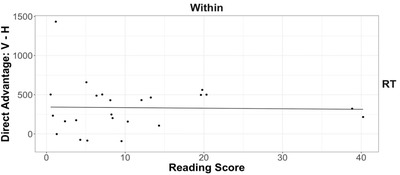
Results of study 1: The relationship between 6‐year‐olds’ reading fluency and horizontal advantage for within‐dimension response time. Each data point represents a participant. The best‐fitting line was estimated by linear regression.

To assess the relationship between reading fluency and horizontal advantage, we first obtained the impedance cost for within‐dimension trials (i.e., RT for impeded placement minus RT for direct placement) for horizontal and vertical triplets for each child, as described above. We then subtracted the impedance cost for horizontal triplets from that for vertical triplets. A positive score means a lower horizontal impedance cost, because it indicates that the direct‐over‐impeded advantage is smaller for horizontal than for vertical triplets. Using the horizontal advantage as the dependent measure, we ran a linear regression with composite reading score as the predictor (Fig. 10). Reading skills failed to predict the degree of horizontal advantage, *β =* −0.72, *t* = −0.11, *p* = .91, *ηp* ^2^ < 0.001. Thus, we find no support for the idea that reading fluency contributes to a lower impedance cost for horizontal figures among 6‐year‐olds.

### 2.3 Study 1 discussion

The results from study 1 are consistent with the idea that visual comparison in 6‐year‐olds follows the structural alignment process and is facilitated by spatial alignment. Our first hypothesis, that the spatial alignment principle applies to even 6‐year‐olds, was confirmed. Children were faster and more accurate for direct placement than for impeded placement. Evidence also confirmed our second hypothesis, that structural alignment should be easier when both object correspondences and relational correspondences are present, compared to when correspondences are purely relational. Here, 6‐year‐olds were faster (and no less accurate) on within‐dimension (object+relation) trials than on cross‐dimension (relation‐only) trials.

For our third hypothesis, we predicted an interaction between placement and dimensionality. This is because objects are highly salient in structural alignment process. This means that in direct placement, objects should add another basis (besides relations) for a same‐different judgment, but in impeded placement, object mismatches should pose another source of interference in arriving at the correct correspondences. The current results failed to provide statistical support for this hypothesis. For both response time and error rate, the direct‐over‐impeded advantage for the within‐dimension trials was not significantly greater than that for the cross‐dimension trials.

We also had two exploratory questions regarding the horizontal advantage. Our first question was whether 6‐year‐olds, like adults, would show a horizontal advantage (i.e., a lower impedance cost for response time for horizontal figures than for vertical figures). The evidence was inconclusive: the direct‐over‐impeded advantage in response time was smaller for horizontal figures on within‐dimension trials (but not on cross‐dimensional trials); but because we saw the opposite (nonsignificant) pattern for error rates, we cannot rule out the possibility of a speed‐accuracy tradeoff where children might have sacrificed accuracy in the interest of speed. Nonetheless, we went on to explore our second question—whether the degree of horizontal advantage would correlate with reading ability. A regression analysis failed to show a significant relationship.

An important overarching question in this research concerns how these processes develop over age and experience. Do the patterns found among 6‐year‐olds also hold for 8‐year‐olds? And do some patterns change as a result of greater experience? In study 2, we tested the same set of hypotheses and questions in 8‐year‐olds. We hypothesized continuity, in that visual comparison in 8‐year‐olds should also follow the structural alignment process and should be facilitated by spatial alignment. However, we also predicted change: we expected the older group to show a higher horizontal advantage, given they should have more experience in reading. We also asked whether this potentially stronger effect of horizontal advantage would be correlated with children's reading fluency.

## 3 Study 2

### 3.1 Methods

#### 3.1.1 Participants

Twenty‐nine 8‐year‐old children participated (*M* = 8.08 years, *Range =* 7.51–8.81 years, 15 females; among the families who indicated race, 79% were White, 5% were Asian, 10% were multiracial, and 5% indicated other races and ethnicities). For the 21 families who indicated parental educational level, all except one indicated college degree or higher. Children were recruited through a database of families from a large Midwestern city who had expressed interest in participating in child research. All families had English as the primary language.

#### 3.1.2 Materials, design, and procedure

All materials, design, and procedure were the same as in study 1, except for one change: 8‐year‐olds received two extra reading assessments at the very end. During the study, they first completed the identical same‐different judgment task on the computer. In the reading assessment phase, they did the same WRF and ORF assessments, respectively, intended for first‐grade students. Using the same assessments as in study 1 ensures that the scores and analyses are directly comparable between younger and older children. However, because these assessments might not accurately capture the reading skills of 8‐year‐olds, we also included two extra reading assessments, the WRF and ORF tasks intended for third‐grade students. For our reading analysis, we separately analyzed the relationship between reading fluency and horizontal advantage using the first‐grade and third‐grade materials. Twenty‐three of the 29 participants completed the four reading assessments.

### 3.2 Results

For the main analyses, data of a block were excluded if participants did not pass the practice within three tries or failed catch trials for that block. Using the same criteria as in study 1, we found only one participant failed the catch trials. We also excluded any trial whose response time was three standard deviations higher than the mean response time. This constituted 1.6% of all trials. Response time was only analyzed for correct trials.

**Fig. 11:**
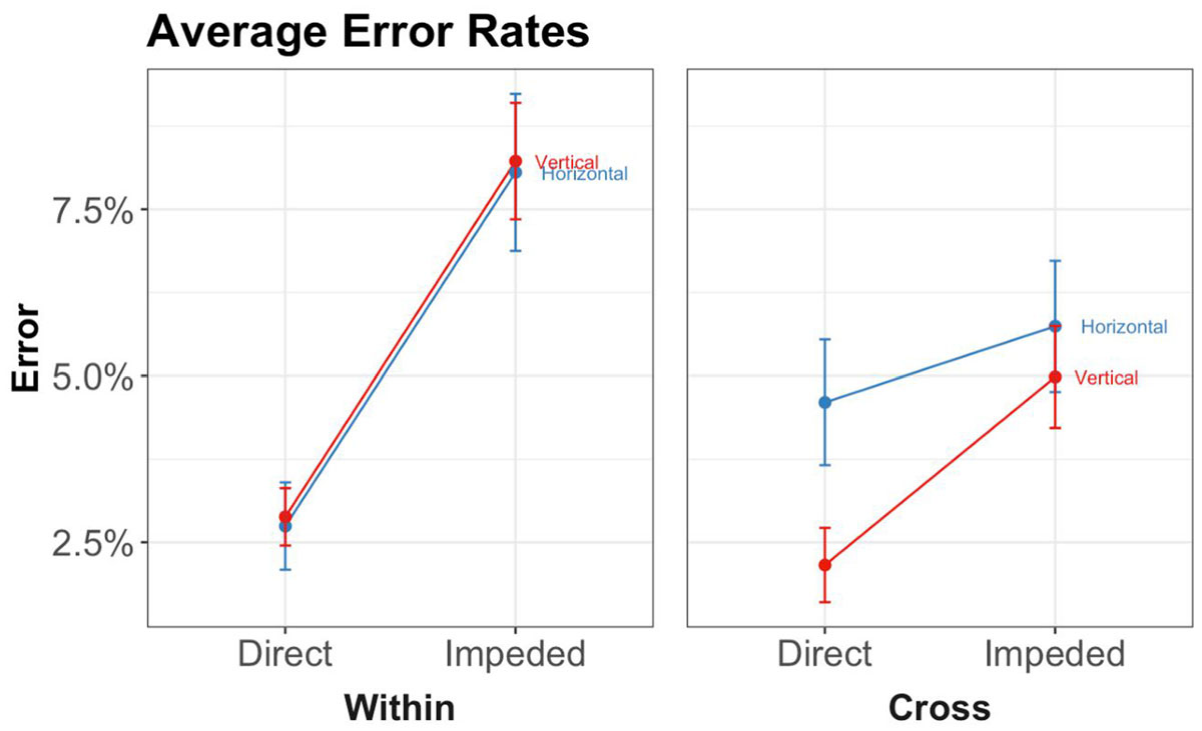
Results of study 2: Error rates by triplet orientation, placement, and whether the block is within‐dimension or cross‐dimension. Error bars represent within‐subject standard errors.

**Fig. 12:**
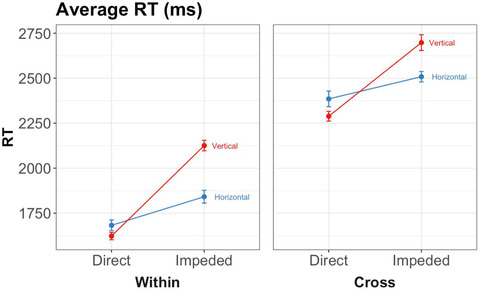
Results of study 2: Response times by triplet orientation, placement, and whether the block is within‐dimension or cross‐dimension. Error bars represent within‐subject standard errors.

To preview, our first two predictions were again borne out: (1) 8‐year‐olds were faster and more accurate on direct placement than on impeded placement, and (2) children were faster on within‐dimension trials than on cross‐dimension trials (see Table 4, and Figs. 11 and 12). Before presenting the planned comparisons testing these predictions, we first present an overall analysis that explores other potential effects.

**Table 4:** Results of study 2: Means and standard deviations for error rates and response times on test trials by dimensionality and placement

		Error rates		RT (in ms)	
Dimensionality	Placement	*M*	*SD*	*M*	*SD*
Within	Direct	2.80%	0.06	1652	438
	Impeded	8.17%	0.11	1978	652
Cross	Direct	3.38%	0.07	2345	832
	Impeded	5.33%	0.11	2605	1047

#### 3.2.1 Effects of spatial alignment and dimensionality

##### Error rates

The repeated‐measures ANOVA on error rates revealed significant main effects of placement, *F*(1, 420) = 20.83, *p* < .001, *ηp* ^2^ = 0.05, and concordance, *F*(1, 420) = 7.69, *p* = .01, *ηp* ^2^ = 0.02. The main effect of dimensionality was not significant, *F*(1, 420) = 1.99, *p* = .16, *ηp* ^2^ = 0.005. There was a significant interaction between placement and dimensionality, *F*(1, 420) = 4.52, *p* = .03, *ηp* ^2^ = 0.01. We report the effect of concordance and the interaction in Supplementary Materials.

Two planned paired *t*‐tests were carried out for our first two predictions. First, we found evidence for the advantage of direct placement—children made significantly more errors on impeded trials (*M* = 0.07, *SD* = 0.11) than on direct trials (*M* = 0.03, *SD* = 0.07), *t* = 4.60, *p* < .001, *d* = 0.40, 95% CI = 0.22–0.58. Our second prediction, that performance would be better on within‐dimension trials than on cross‐dimension trials, was not borne out. There was no significant difference in error rates between cross‐dimension trials (*M* = 0.04, *SD* = 0.09) and within‐dimension trials (*M* = 0.05, *SD* = 0.09), *t* = −1.49, *p* = .10, *d* = −0.12, 95% CI = −0.28 to 0.04.

##### Response time

The repeated‐measures ANOVA on response times revealed main effects of placement, *F*(1, 420) = 55.82, *p* < .001, *ηp* ^2^ = 0.12, dimensionality, *F*(1, 420) = 284.03, *p* < .001, *ηp* ^2^ = 0.40, and concordance, *F*(1, 420) = 7.58, *p* = .01, *ηp* ^2^ = 0.02. There was also a significant interaction between placement and orientation, *F*(1, 420) = 15.60, *p* < .001, *ηp* ^2^ = 0.04. We report the effect of concordance and the interaction in Supplementary Materials.

Planned paired *t*‐tests showed that, consistent with Prediction 1, children were faster on direct trials (*M* = 1999, *SD* = 749) than on impeded trials (*M* = 2291, *SD* = 925), *t* = 8.98, *p* < .001, *d* = 0.33, 95% CI = 0.25–0.40. Results were also consistent with Prediction 2: children were faster on within‐dimension trials (*M* = 1815, *SD* = 577) than on cross‐dimension trials (*M* = 2475, *SD* = 952), *t* = 15.27, *p* < .001, *d* = 0.73, 95% CI = 0.63–0.84. Together with the error rates, the results supported our first hypothesis: 8‐year‐olds, like 6‐year‐olds, were both faster and more accurate on direct placement than on impeded placement (Prediction 1). The results were also consistent with our second hypothesis: children were faster on within‐dimension trials than on cross‐dimension trials (with no evidence of a speed‐accuracy tradeoff) (Prediction 2).

#### 3.2.2 Interaction between spatial alignment and dimensionality

**Fig. 13:**
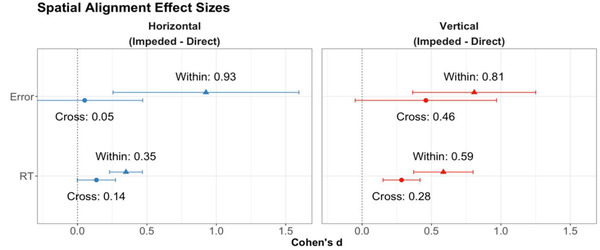
Results of study 2: Effect sizes for error rates and response times by triplet orientation, placement, and whether the block is within‐dimension or cross‐dimension. Error bars represent confidence intervals.

Our third hypothesis was that the advantage of direct over impeded placement would be greater for within‐dimension trials than for cross‐dimension trials. To test this, we compared the effect sizes of error rates and response times between direct and impeded placement and did this for both within‐ and cross‐dimension trials. As for study 1, we combine horizontal and vertical figures for this analysis, but present separate graphs for the two orientations for clarity. Table 5 and Fig. 13 summarize the statistics.

**Table 5:** Results of study 2: Effect size estimates for the advantage of direct over impeded placement in 8‐year‐olds

	Within		Cross	
Measure	*d*	*95% CI*	*d*	*95% CI*
Error rates	1.04	0.44, 1.64	0.26	−0.18, 0.70
RT	0.41	0.30, 0.53	0.21	0.09, 0.32

For both error rates and response times, Cohen's *d*s were not significantly larger for the within‐dimension trials than for the cross‐dimension trials—a pattern that parallels the findings for the 6‐year‐olds. A combined analysis over both age groups revealed significantly larger effect sizes for direct placement over impeded placement for within‐dimension than for cross‐dimension trials. This held for both error rates and response times, providing support for our third hypothesis (Table 6).

**Table 6:** Results of study 2: Effect size estimates for the advantage of direct over impeded placement in 6‐ and 8‐year‐olds combined

	Within		Cross	
Measure	*d*	*95% CI*	*d*	*95% CI*
Error rates	0.80	0.47, 1.12	0.24	0.06, 0.42
RT	0.37	0.28, 0.45	0.18	0.12, 0.24

#### 3.2.3 Horizontal advantage

Our exploratory questions concerned with whether 8‐year‐olds show a horizontal advantage—that the processing cost of impeded placement is lower for horizontal figures than for vertical figures—and whether this would be related to reading fluency. In study 1, we did not find evidence of a horizontal advantage among 6‐year‐olds. However, if Matlen et al. (2020) are correct that reading practice leads to robust horizontal encoding, we would expect 8‐year‐olds to show a larger horizontal advantage due to more reading practice.

The horizontal advantage was measured by first subtracting the response times of direct trials from those of impeded trials. A positive score indicates the presence of an impedance cost (in other words, a direct‐over‐impeded advantage). This difference score was calculated for triplets of both horizontal and vertical orientations. If 8‐year‐olds mirror the adults’ pattern, they will show a lower impedance cost in response time for horizontal than for vertical figures (i.e., a horizontal advantage). Paired *t*‐tests showed a horizontal advantage in impedance cost for both within‐dimension trials (*M_Horizontal_* = 162, *SD_Horizontal_* = 144 vs. *M_Vertical_* = 474, *SD_Vertical_* = 430), *t* = 3.68, *p* < .001, *d* = 0.97, 95% CI = 0.33–1.62, and cross‐dimension trials (*M_Horizontal_* = 135, *SD_Horizontal_* = 364 vs. *M_Vertical_* = 405, *SD_Vertical_* = 500), *t* = 3.54, *p* = .001, *d* = 0.60, 95% CI = 0.23–0.97. Thus, 8‐year‐olds, like adults and unlike 6‐year‐olds, show a significant horizontal advantage in response time—a lower impedance cost for horizontal figures than for vertical figures. Importantly, we did not see evidence of a speed‐accuracy tradeoff for 8‐year‐olds: *t*‐tests showed that the impedance cost for error rates did not differ between horizontal and vertical triplets for within‐dimension trials (*M_Horizontal_* = 0.052, *SD_Horizontal_* = 0.085 vs. *M_Vertical_* = 0.050, *SD_Vertical_* = 0.064), *p* = .90, nor for cross‐dimension trials (*M_Horizontal_* = 0.004, *SD_Horizontal_* = 0.083 vs. *M_Vertical_* = 0.028, *SD_Vertical_* = 0.080), *p* = .09. In fact, for cross‐dimension trials, the impedance cost for error rate was nonsignificantly *lower* for horizontal than for vertical triplets; thus, we can rule out a speed‐accuracy tradeoff for this condition. These findings provide support for the idea that the horizontal advantage increases over age and experience.

#### 3.2.4 Horizontal advantage and reading fluency

Last, we ask whether the horizontal advantage in response time is related to reading fluency. To do so, we regress the degree of horizontal advantage on children's reading ability, as assessed by the four standard reading tests. We report 8‐year‐olds’ performance both using the first‐grade reading materials (so that the results are directly comparable across ages) and the age‐appropriate third‐grade materials.

For the first‐grade materials, on average, 8‐year‐old children read 67.30 (*SD* = 20.55) correct words in the WRF test and 130.61 (*SD* = 40.39) correct words in the ORF test (see Appendix B). As expected, 8‐year‐olds outperformed 6‐year‐olds in the first‐grade reading materials for both WRF and ORF, both *ps* < .001. Performance on the two tests was highly correlated, *r* = .76, *p* < .001, so we combined the WRF and ORF scores into a composite score using the weights intended for first‐grade materials [WRF * 0.1351 + ORF * 0.2536]. The resulting reading score had a mean of 42.22 and a standard deviation of 12.47.

**Fig. 14:**
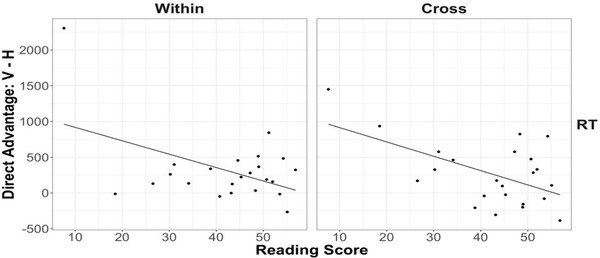
Results of study 2: The relationship between 8‐year‐olds’ reading fluency (using the first‐grade materials) and horizontal advantage for within‐ and cross‐dimension response time. Each data point represents a participant. The best‐fitting lines were estimated by linear regression.

We computed the degree of horizontal advantage for each child by subtracting the [Impeded – Direct] difference score in response time for horizontal triplets from that for vertical triplets. We did this for both within‐ and cross‐dimensions because 8‐year‐olds showed a horizontal advantage on both trial types. To explore the relationship between reading fluency and horizontal advantage, we ran two linear regressions with the horizontal advantage as the dependent measure and the composite reading score as the predictor (Fig. 14). The regressions showed that surprisingly, greater reading skills in fact were associated with a *higher* impedance cost for horizontal figures on both within‐dimension trials, *β =* −18.76, *t* = −2.47, *p* = .02, *ηp* ^2^ = 0.22, and cross‐dimension trials, *β = −*20.04, *t* = −3.06, *p* = .01, *ηp* ^2^ = 0.31. This was opposite to what we expected.

**Fig. 15:**
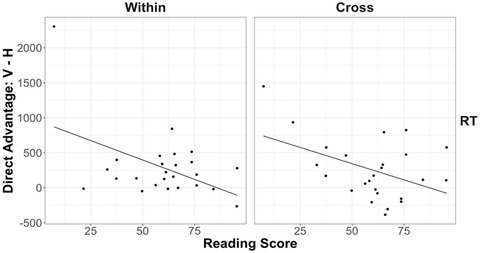
Results of study 2: The relationship between 8‐year‐olds’ reading fluency (using the third‐grade materials) and horizontal advantage for within‐ and cross‐dimension response time. Each data point represents a participant. The best‐fitting lines were estimated by linear regression.

For the third‐grade materials, on average, 8‐year‐old children read 60.34 (*SD* = 19.94) correct words in the WRF test and 118.74 (*SD* = 44.91) correct words in the ORF test (see Appendix C). Performance on the two reading scales was highly correlated, *r* = .83, *p* < .001, so the WRF and ORF scores were combined into a composite score using the weights intended for third‐grade materials [WRF * 0.1983 + ORF * 0.3942] (DIBELS Scoring Guide, 2019). The resulting reading score had a mean of 58.77 and a standard deviation of 21.11. We ran two linear regressions with the composite reading score as the predictor and the degree of horizontal advantage as the dependent variable (Fig. 15). We again found a pattern opposite to our prediction: Greater reading skills were associated with a *higher* impedance cost for horizontal figures on both within‐dimension trials, *β =* −11.05, *t* = −2.46, *p* = .02, *ηp* ^2^ = 0.22, and cross‐dimension trials, *β = −*9.55, *t* = −2.29, *p* = .03, *ηp* ^2^ = 0.20. Overall, we find no support for the claim that reading fluency contributes to a horizontal advantage.

##### 3.2.4.1 Regression between reading and horizontal advantage across 6‐ and 8‐year‐olds

**Fig. 16:**
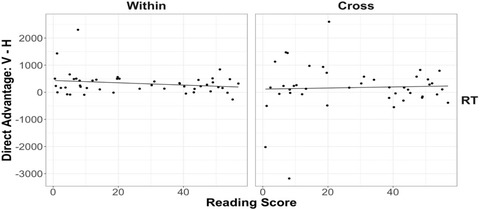
The relationship between reading fluency and horizontal advantage for within‐ and cross‐dimension response time for 6‐ and 8‐year‐olds. Each data point represents a participant. The best‐fitting lines were estimated by linear regression.

As discussed above, our sample sizes for the individual age groups (*n* = 29 for each age) were planned based on our main within‐subject predictions for spatial alignment. But to adequately test the between‐subject relationship between reading ability and performance (i.e., the horizontal advantage), a sample size of at least 44 is required. In order to achieve the necessary power for a between‐subject analysis, we combined the two age groups, using the same first‐grade reading assessment for both groups. This gave us a sufficient sample size (48 participants who completed the reading assessments) to test the correlation between reading ability and the degree of horizontal advantage in response time. The regression analyses showed that reading skills failed to predict the degree of horizontal advantage for both within‐dimension trials, *β =* −4.21, *t* = −1.34, *p* = .18, *ηp* ^2^ = 0.04, and cross‐dimension trials, *β =* 2.00, *t* = 0.28, *p* = .78, *ηp* ^2^ = 0.002. Fig. 16 depicts the results. Thus, although we find evidence for a horizontal advantage in 8‐year‐olds, we do not find evidence (either within 8‐year‐olds or within the combined 6‐ and 8‐year‐olds) that reading fluency is related to this advantage. We return to this issue in the General Discussion.

## 4 General discussion

We began with three predictions. First, we predicted that both 6‐ and 8‐year‐old children's performance would follow the spatial alignment principle (Matlen et al., 2020). That is, children should be faster and/or more accurate in visual comparison for direct placement—when spatially corresponding elements and relations are juxtaposed and far from competing matches—than for impeded placement. Importantly, consistent with structure‐mapping theory, this prediction should hold both for within‐dimension (overall similarity) comparisons and for cross‐dimension (purely relational) comparisons. Our second prediction, also following structure‐mapping theory, is that children's performance should be better on within‐dimension (object+relation) trials than on cross‐dimension (relation‐only) trials. We found strong support for both of these predictions in both 6‐ and 8‐year‐olds. Our third prediction was that the advantage of direct over impeded placement would be larger for object+relation trials than for relation‐only trials. Support for this prediction was found in a combined analysis across the two age groups, although not in the individual age‐group analyses. We now consider these findings in more detail.

*Prediction 1: Spatial alignment*. Across two studies, the research provides evidence that visual comparison among 6‐ and 8‐year‐old children follows the spatial alignment principle (Matlen et al., 2020). According to this, visual comparison is most efficient when the figures to be compared are shown in direct placement—that is, when the figures are juxtaposed with their structural axes parallel, and with placement orthogonal to the structural axes of the figures. This placement maximizes the ease of achieving a structural alignment between the two figures and minimizes the influence of potential competing correspondences. There were two key results that supported this idea. First, both 6‐ and 8‐year‐old children were faster and more accurate for direct placement than for impeded placement. Second, the advantage of direct placement held for both within‐dimension (object+relation) matches and cross‐dimension (purely relational) matches, showing that, as in adults' comparison, structural alignment can occur with purely relational pairs—object matches are not required.

*Prediction 2*. Although structural alignment can occur with purely relational matches, the process is easier if the objects also match. Thus, our second prediction was that children would be faster and/or more accurate on within‐dimension (object+relation) trials than on cross‐dimension (relation‐only) trials. This prediction too was borne out—both age groups were faster (and importantly, no less accurate) for the within‐dimension trials than for the cross‐dimension trials. This is consistent with structure‐mapping theory (Forbus et al., 2017; Gentner & Forbus, 2011; Gentner, 1983, 2010; Markman & Gentner, 1993a; Sagi et al., 2012), which holds that although people rely on the same mechanism for purely relational and concrete matches, overall similarity—in which the object matches support the relational alignment—is easier to process. In overall similarity, object matches and relational matches support the same set of mappings, thereby facilitating the alignment process. (Forbus et al., 2017; Gentner & Forbus, 2011; Gentner & Kurtz, 2006; Lovett, Gentner, Forbus, & Sagi, 2009). This pattern holds both for perceptual comparisons (e.g., Goldstone, 1994; Sagi et al., 2012) and for conceptual comparisons (Gentner & Kurtz, 2006). For example, Gentner and Kurtz (2006) asked adults to rate the analogical relatedness of sentence pairs, which varied in their degree of object and relational similarities. The results showed that (1) analogy judgment was dominated by relational similarity, but (2) processing time was faster when both objects and relations were similar. Thus, for conceptual comparison, as for perceptual comparison, overall similarity is easier to process than purely relational matches, consistent with structure‐mapping theory. Finding this pattern in our data provides further support for the idea that children's visual comparison involves a process of structural alignment, and that this process is facilitated by spatial alignment.

*Prediction 3*. Our third prediction was that the advantage of direct over impeded placement would be larger for object+relation trials than for relation‐only trials, as for Matlen et al.’s results. This is because object matches—which are highly salient both for children and adults (Gentner & Kurtz, 2006; Gentner & Toupin, 1986; Goldstone & Medin, 1994)—should facilitate alignment in direct placement, but should hinder it in impeded placement, by posing potential intervening object matches. We found support for this prediction in an analysis combining the data of 6‐ and 8‐year‐olds. The effect sizes for the direct‐over‐impeded advantage were significantly larger for the object+relation (within‐dimension) trials than for the relation‐only (cross‐dimension) trials. This held for both error rate and response time, mirroring the patterns found in adults (Matlen et al., 2020). (We note that within each age the patterns were consistent with the prediction but were not significant.) This result provides further support to the idea that visual comparison follows a structural alignment process.

### 4.1 Horizontal advantage and reading fluency

The main finding of the present study was that the spatial alignment process holds for visual comparison in both 6‐ and 8‐year‐old children. This suggests continuity in the development of visual comparison process from middle childhood to adulthood. A secondary question was to explore whether the effects of spatial alignment are influenced by experience. In particular, Matlen et al. (2020) found that adults showed a horizontal advantage in response time—that is, the increase in response time for impeded placement (relative to that for direct placement) was lower for horizontal figures than for vertical figures. Matlen et al. speculated that this horizontal advantage might be due to greater horizontal encoding fluency. They further conjectured that a major contributor to horizontal fluency could be reading experience, given the horizontal pattern of writing in English (see Thibaut et al., 2010). If so, this pattern should be absent or diminished in children, who have far less reading experience. Therefore, this study explored whether children with different levels of reading experience would show evidence of a horizontal advantage.

Two predictions follow if Matlen et al.’s hypothesis was correct. First, horizontal advantage (if found) should be larger in 8‐year‐olds, who have more experience in reading, than in 6‐year‐olds. Second, the degree of horizontal advantage should be related to reading proficiency. Our findings supported the first prediction: We failed to find evidence of a horizontal advantage in 6‐year‐olds, but 8‐year‐olds showed a strong horizontal advantage in response time for both object+relation and relation‐only matches. The second prediction was tested by regressing the degree of horizontal advantage on children's reading ability. The results failed to support the claim that reading contributes to more fluent horizontal encoding. Indeed, we found an opposite pattern in 8‐year‐olds.

Given our evidence of an experience‐based horizontal advantage, one possibility is that this advantage arises from more general experience with patterns in the world, such as frequent exposure to horizontally symmetric faces. Alternately, it may be that reading experience does contribute but that fluency takes longer to develop. Future research should continue to explore possible effects of reading proficiency, as well as other pathways to horizontal fluency.

### 4.2 Implications and open questions

These findings bear on the well‐supported generalization that spatiotemporal proximity facilitates visual comparison (Christie & Gentner, 2010; Jee et al., 2013; Kok et al., 2013; Namy & Gentner, 2002). Researchers have proposed that spatiotemporal juxtaposition is effective because having the analogs copresent encourages learners to compare them (Christie & Gentner, 2010) and reduces the cognitive processing load in carrying out comparison (Begolli et al., 2018). However, the current findings suggest a further consideration: the advantage of spatiotemporal juxtaposition may stem in part from spatial alignment. In many prior studies, the visual figures were optimally placed in terms of the spatial alignment principle. Future research should examine the degree to which high spatial alignment contributes to the observed gain from spatiotemporal proximity.

One limitation of the current study is that the cross‐dimension block always followed the within‐dimension blocks. This could have affected children's performance on the cross‐dimension trials. Because children had the opportunity to engage in within‐dimension pattern matching before the more difficult cross‐dimension matching task, this could have facilitated children's performance on the later purely relational matches. This is consistent with the idea of progressive alignment (Kotovsky & Gentner, 1996). That is, the relatively easy within‐dimension matches (in which the object similarities support the relational alignment) could have highlighted the common relational structure and facilitated the more distant cross‐dimension comparisons. Thus, our findings might have inflated children's cross‐dimension performance. On the other hand, children might also have been more fatigued toward the end of the experiment, contributing to their lower accuracy and speed on the cross‐dimension trials. Future studies should counterbalance the order of block dimensionality to address these concerns.

Another limitation is that the current study focused on simple spatial patterns with a clear structural axis. A key issue is how well the spatial alignment principle will scale up to more complex spatial patterns. There is some encouraging research in this direction. Simms et al. (2020) showed seventh grade children pairs of skeletons and asked them to identify an incorrect bone in one structure by comparing it with a correct model (adapting the method from Kurtz and Gentner, 2013). The results indicated that when the figures were in noncanonical orientation and thus more difficult for students, direct placement facilitated error detection. This is consistent with the idea that spatial alignment may be especially effective when learners are less able to apply existing knowledge to guide encoding. In sum, it appears that the spatial alignment principle applies in processing rich spatial patterns as well as simple ones. This brings us to its use in designing educational materials.

### 4.3 Implications for education

**Fig. 17:**
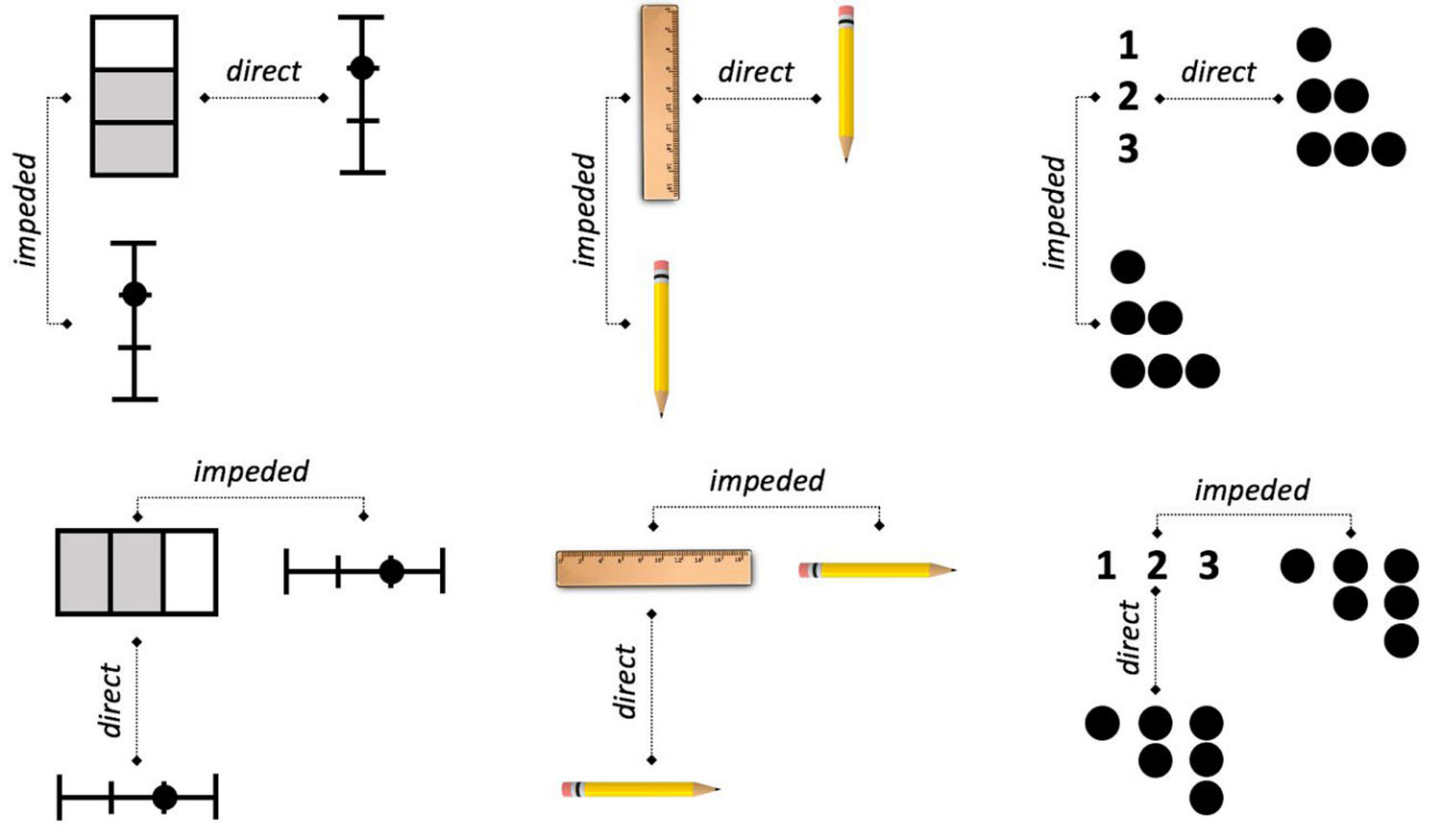
Suggested uses of spatial alignment in education, depicting direct and impeded placements for figures with vertical axes (top row) and horizontal axes (bottom row). From left to right: Fraction representations, ruler and pencil comparison, and numeric symbols and their corresponding quantities.

Visualizations are prevalent in education and important for students’ learning (Ainsworth et al. 2011; Forbus et al., 2011; Gattis & Holyoak, 1996; Jee et al., 2019, 2013; Kellman et al., 2010; Mayer, 1993; Novick, 2001; Schunn et al., 2018; Uttal, Fisher, & Taylor, 2006). A particularly useful strategy is to present pairs of figures that students can compare to grasp underlying commonalities and differences (Christie & Gentner, 2010; Gentner & Namy, 1999; Matlen et al., 2011; Rittle‐Johnson & Star, 2009; Vendetti et al., 2015). Through comparison, abstract conceptual relations are more likely to be encoded and transferred to new cases (Christie & Gentner, 2010; Loewenstein & Gentner, 2005; Rittle‐Johnson & Star, 2007; Schwartz et al., 2011). For example, in mathematics, students in first grade are expected to compare lengths of objects using a common unit of measurement (Common Core 1.MD.A.1). Young children perform better in these tasks when the object to be measured is spatially aligned with the ruler (Solomon et al. 2015). Fig. 17 shows our suggestions for ways in which spatial alignment may be useful in education.

Comparison highlights similarities and also differences that are connected to a shared system of relations (Goldwater & Gentner, 2015; Kurtz et al., 2001; Rittle‐Johnson & Star, 2007; Star & Rittle‐Johnson, 2009). In the context of science learning, contrasting examples can enable learners to zero in on conceptually relevant details, such as geological structures (Jee et al. 2014, 2013; Matlen et al. 2011), lung diseases in X‐rays (Kok et al., 2013), anomalies in skeletons (Kurtz & Gentner, 2013), and building features that reflect elementary engineering principles (Gentner et al., 2016; Hoyos & Gentner, 2017), to list a few examples.

Yet, despite the potential advantages of visual comparison, recent research on the presentation of visual pairs in textbooks has found wide variation in the effectiveness of this presentation (Jee, Matlen, Gentner, & Simms, 2019: Jee, Matlen, Simms, & Gentner, under review). It appears that middle‐school science and mathematics texts often fail to present visual comparisons in optimal ways. Ongoing work from our group is assessing the effect of spatial alignment in classroom learning using a wide range of textbook materials (Jee et al., under review). Articulating the principles of design for visual comparison can inform educational practice and instructional design to better support students’ learning.

## 5 Conclusion

We found that the spatial alignment principle held for both 6‐ and 8‐year‐olds, showing continuity in visual comparison processing across development. These results support the idea that visual comparison involves a process of structural alignment. Apart from their implications for basic visual processes, the results are relevant for learning and instruction. Attention to the spatial alignment principle and other principles of visual comparison could have important benefits for the design of instructional materials.

## Supporting information




**Table S1**: Effect size estimates for response time in 6‐year‐olds after excluding fast trials.
**Table S2**: Effect size estimates for response time in 6‐ and 8‐year‐olds using *MAD*.

## Notes

1 It is important to note that object matches facilitate structural alignment when similar objects play the same roles in the common relational structure (overall similarity). When similar objects play different roles in the common structure, as in cross‐mapping, structural alignment is hindered. The competing object matches may prevent learners from perceiving the underlying relational commonalities (Gentner & Toupin, 1986; Loewenstein & Gentner, 2005; Markman & Gentner 1993a,b; Richland, Morrison, & Holyoak, 2006; Murphy et al., 2021).

2 In fact, in Begolli and Richland (2016), children given sequential presentation performed worse than a group who saw no visuals at all. The sequential presentation group may have failed to carry out structural alignment, instead retaining the details of the presented items (one of which showed a misconception).

3 It is possible that in some cases, there may be more than one structural axis for a given figure or a pair of figures. For simplicity, we restrict our current account to cases where there is a clear axis.

4 We note that children's performance on the cross‐dimension (relation‐only) trials may have benefited from the task order, where the cross‐dimension trials always followed the within‐dimension (object+relation) trials. This provides the opportunity for progressive alignment, as discussed earlier, and could bias against Prediction 2. However, we believed (correctly, as it turned out) that such training would not erase the inherently greater difficulty of the cross‐dimension trials.

5 We also analyzed response time data by excluding responses faster than 200 ms and by using absolute deviation around the median (MAD) instead of three standard deviations above the mean. The same patterns were found, as in the main analyses, with one exception: the 8‐year‐old findings for Prediction 3 were somewhat stronger using MAD. We report this and other ancillary analyses in Supplementary Materials.

6 To plot within‐subject error bars, we removed between‐subject variability by subtracting subject average from individual scores and adding back grand average for each condition (i.e., with respect to dimensionality, placement, and orientation). Standard errors were calculated from the new scores to capture within‐subject variance only (Cousineau, 2005).

7
*Th*‐tests were paired such that in examining the focal effect of interest (e.g., the effect of placement), scores were paired for the focal variable and matched on other variables (e.g., dimension, orientation, and concordance).

8 We see no evidence of a speed‐accuracy tradeoff here—children were nonsignificantly more accurate on within‐dimension than on cross‐dimension trials.

## Appendix A

### A.1

Raw reading assessment scores for 6‐year‐olds (using the first‐grade materials). Each point refers to a participant. Horizontal bars represent medians.

## Appendix B

### B.1

Raw reading assessment scores for 8‐year‐olds (using the first‐grade materials intended for 6‐year‐olds). Each point refers to a participant. Horizontal bars represent medians.

## Appendix C

### C.1

Raw reading assessment scores for 8‐year‐olds (using the age‐appropriate third‐grade materials). Each point refers to a participant. Horizontal bars represent medians.
