# Supplementary material for: Spatial Alignment Facilitates Visual Comparison in Children
Source: Cogn Sci. 2022 Aug 16;46(8):e13182. doi: 10.1111/cogs.13182 (PMC9540866; doi:10.1111/cogs.13182)
Supplement: Supplementary file 1 — Table S1: Effect size estimates for response time in 6‐year‐olds after excluding fast trials. Table S2: Effect size estimates for response time in 6‐ and 8‐year‐olds using MAD. [file COGS-46-e13182-s001.docx]

*S1 Response Time*

In the paper we analyzed our response time data after excluding any trial whose response time was three standard deviations longer than the mean response time with respect to each subject and block dimensionality. Our main reason for using this criterion is to be compatible with the method used for adults in Matlen et al. (2020). However, to ensure robustness of our findings, below we report the results for response time using two other methods: additionally excluding fast trials and replacing standard deviation with absolute deviation around the median (*MAD*). We reran our analyses for the three main predictions, as they are central to our hypotheses. To preview, the same patterns were found as in the main paper, despite the different methods, with one exception: the 8-year-old findings for Prediction 3 were somewhat stronger here.

*S1.1 Excluding fast trials*

Besides excluding slow trials (three standard deviations above the mean) as in the main paper, we also excluded fast trials (less than 200ms). Excluding the fast trials led to omitting an additional 0.27% of trials for 6-year-olds. None of 8-year-olds completed trials faster than 200ms. Therefore, we restricted our analyses to only 6-year-olds.

*S1.1.1 Effects of Spatial Alignment and Dimensionality*

The repeated-measures ANOVA on response times revealed main effects of placement, *F*(1, 380) = 11.89, *p* < .001, *ηp2* = .03, and dimensionality, *F*(1, 380) = 201.84, *p* < .001, *ηp2* = .35. Consistent with Prediction 1, planned paired T-tests showed that, 6-year-olds were faster on direct (*M* = 2908, *SD* = 1570) than on impeded trials (*M* = 3227, *SD* = 1690), *t* = 4.67, *p* < .001, *d* = .19, 95% CI = .11-.28. Prediction 2 was also borne out: children were faster on within-dimension trials (*M* = 2488, *SD* = 757) than on cross-dimension trials (*M* = 3716, *SD* = 2062) blocks, *t* = 11.66, *p* < .001, *d* = .71, 95% CI = .58-.85.

*S1.1.2 Interaction between Spatial Alignment and Dimensionality*

Prediction 3 is that the direct advantage should be greater for within-dimension than for cross-dimension trials. After excluding fast trials for response times, we found that for 6-year-olds, Cohen’s ds continued to appear larger for within-dimension trials than for cross-dimension trials. However, the overlap in confidence intervals suggests that the differences between trial types were not significant.

Table S1. effect size estimates for response time in 6-year-olds after excluding fast trials.

|  |  | Within | | | |  | | Cross | |
| --- | --- | --- | --- | --- | --- | --- | --- | --- | --- |
| Measures |  | | *d* | *95% CI* |  | | *d* | | *95% CI* |
|  |  | |  |  |  | |  | |  |
| RT |  | | 0.31 | 0.18, 0.44 |  | | 0.19 | | 0.11, 0.26 |

*S1.2 Using absolute deviation around the median (MAD)*

Instead of using standard deviation, we used *MAD* as an alternative method to exclude slow trials because response time may have a skewed distribution. MAD, unlike standard deviation, is a robust measure of central tendency and not sensitive to the presence of outliers. We excluded trials that were 3 MADs above the median with respect to each subject and block dimensionality. This constituted 5.82% trials for 6-year-olds and 5.48% for 8-year-olds. (We note that fast trials were not excluded here).

*S1.2.1 Effects of Spatial Alignment and Dimensionality*

*6-year-olds.* The repeated-measures ANOVA on response times revealed main effects of placement, *F*(1, 380) = 9.31, *p* = .002, *ηp2* = .02, and dimensionality, *F*(1, 380) = 205.04, *p* < .001, *ηp2* = .30. Consistent with Prediction 1, planned paired T-tests showed that 6-year-olds were faster on direct (*M* = 2785, *SD* = 1461) than on impeded trials (*M* = 3037, *SD* = 1499), *t* = 3.84, *p* < .001, *d* = .17, 95% CI = .08-.26. Consistent with Prediction 2, children were also faster on within-dimension trials (*M* = 2398, *SD* = 702) than on cross-dimension trials (*M* = 3487, *SD* = 1870) blocks, *t* = 11.66, *p* < .001, *d* = .72, 95% CI = .58-.86.

*8-year-olds.* The repeated-measures ANOVA on response times revealed main effects of placement, *F*(1, 420) = 46.66, *p* < .001, *ηp2* = .10, and dimensionality, *F*(1, 420) = 305.16, *p* < .001, *ηp2* = .42. Consistent with Prediction 1, planned paired T-tests showed that 8-year-olds were faster on direct (*M* = 1946, *SD* = 731) than on impeded trials (*M* = 2194, *SD* = 872), *t* = 9.18, *p* < .001, *d* = .29, 95% CI = .23-.36. Consistent with Prediction 2, children were also faster on within-dimension trials (*M* = 1753, *SD* = 515) than on cross-dimension trials (*M* = 2388, *SD* = 920), *t* = 15.12, *p* < .001, *d* = .72, 95% CI = .61-.82.

*S1.2.2 Interaction between Spatial Alignment and Dimensionality*

As in the main paper, we found no evidence for this prediction in 6-year-olds using three MADs above the median: although Cohen’s ds appeared larger for within-dimension trials than for cross-dimension trials, the overlap in confidence intervals suggests nonsignificance. In contrast, for 8-year-olds, results were somewhat stronger than in the main paper: the difference in the direct-over-impeded advantage was significantly larger for within-dimension trials than for cross-dimension trials. This pattern also held when data were combined for 6- and 8-year-olds, thereby yielding some support for our third prediction (see Table S2).

Table S2. effect size estimates for response time in 6- and 8-year-olds using *MAD*.

|  |  |  | Within | | | |  | | Cross | |
| --- | --- | --- | --- | --- | --- | --- | --- | --- | --- | --- |
| Group | Measure |  | | *d* | *95% CI* |  | | *d* | | *95% CI* |
|  |  |  | |  |  |  | |  | |  |
| 6-year-olds | RT |  | | 0.33 | 0.20, 0.45 |  | | 0.14 | | 0.05, 0.23 |
| 8-year-olds |  |  | | 0.43 | 0.29, 0.58 |  | | 0.17 | | 0.08, 0.26 |
| 6- and 8-year-olds |  |  | | 0.35 | 0.27, 0.44 |  | | 0.16 | | 0.10, 0.23 |

*S2 Other ANOVA Effects in Study 2*

*Error rates*. In Study 2, our overall ANOVA on error rates revealed a significant main effect of concordance, *F*(1, 420) = 7.69, *p* = .01, *ηp2* = .02, and a significant interaction between placement and dimensionality, *F*(1, 420) = 4.52, *p* = .03, *ηp2* = .01. A follow-up paired t-test with Bonferroni correction explored the effect of concordance: 8-year-olds made more errors on “same” (*M* = 0.06, *SD* = 0.11) than on “different” (*M* = 0.04, *SD* = 0.07) trials, *t* = 2.89, *p* = .01, *d* = .24, 95% CI = .07-.40.

To explore the interaction between placement and dimensionality, we grouped data by dimensionality (within- or cross-dimension) and compared children’s response times for direct and impeded placement (see Figure 11 and Table 2 in the main paper). For within-dimension trials, children made more errors on impeded placement (*M* = 0.08, *SD* = 0.11) than on direct placement (*M* = 0.03, *SD* = 0.06) after Bonferroni correction, *t* = 5.29, *p* < .001, *d* = .61, 95% CI = .36-.85. For cross-dimension trials, error rates were not significantly different between impeded placement (*M* = 0.05, *SD* = 0.11) and direct placement (*M* = 0.03, *SD* = 0.06) *t* = 1.62, *p* = .33, *d* = .21, 95% CI = -.05 - .46. This finding adds nuance to the results reported in the main paper, which showed that overall children made significantly more errors on impeded (*M* = 0.07, *SD* = 0.11) than on direct (*M* = 0.03, *SD* = 0.07) trials, *t* = 4.60, *p* < .001, *d* = .40, 95% CI = .22-.58. Importantly, as shown in the main paper, children were also faster on direct placement than on impeded placement for both within- and cross-dimension trials. Together, these results are consistent with our Prediction 1: children should be faster and/ or more accurate on direct placement than on impeded placement.

*Response time.* The ANOVA on response times similarly revealed a main effect of concordance, *F*(1, 420) = 7.58, *p* = .01, *ηp2* = .02, and a significant interaction between placement and orientation, *F*(1, 420) = 15.60, *p* < .001, *ηp2* = .04. A follow-up paired t-test showed that children were faster on “same” (*M* = 2091, *SD* = 854) than on “different” (*M* = 2199, *SD* = 851) trials, *t* = -3.56, *p* = .001, *d* = -.13, 95% CI = -.20 - -.06. Together with error rates, the results suggest that 8-year-olds made more errors when the pairs contained the same pattern than when the pairs were different; however, when they responded correctly, they were faster for the “same” pairs (response time was analyzed only for correct trials).

To explore the interaction between placement and orientation, we grouped data by placement and compared children’s response times for horizontal and vertical figures (see Figure 12 in the main paper). On direct trials, response time was not significantly different between horizontal (*M* = 2040, *SD* = 809) and vertical (*M* = 1958, *SD* = 684) triplets after Bonferroni correction, *t* = 2.08, *p* = .12, *d* = .11, 95% CI = .01-.21. On impeded trials, children were faster for horizontal (*M* = 2178, *SD* = 859) than for vertical (*M* = 2405, *SD* = 977) triplets, *t* = -5.42, *p* < .001, *d* = -.24, 95% CI = -.33 - -.15. This suggests that children were faster (and importantly, not less accurate) on impeded trials for horizontal figures than for vertical figures for both within- and cross-dimension trials (see Figure 12 in the paper). We note that this is consistent with the possibility that 8-year-olds have developed a horizontal advantage (as discussed in the body of the paper).

*S3 Preliminary Analysis for Studies 1 & 2*

*Study 1.* Preliminary t-tests were conducted for whether participants saw the shape block or color block first and for whether the “x” key or the “comma” key was labeled as “S.” Results showed that neither factor significantly predicted participants’ accuracy or response time (For which key is labeled as “S”, *p* = 1 for response time and *p* = 1 for error rates; for which within-dimension block appears first, *p* = 1 for response time and *p* = .44 for error rates. All p-values were Bonferroni corrected).

*Study 2.* The same preliminary t-tests were carried out. Results showed that neither factor significantly predicted participants’ accuracy or response time (For which key is labeled as “S”, *p* = .28 for response time and *p* = 1 for error rates; for which within-dimension block appears first, *p* = 1 for response time and *p* = .08 for error rates. All p-values were Bonferroni corrected).
